# System Responses to Equal Doses of Photosynthetically Usable Radiation of Blue, Green, and Red Light in the Marine Diatom *Phaeodactylum tricornutum*

**DOI:** 10.1371/journal.pone.0114211

**Published:** 2014-12-03

**Authors:** Kristin Collier Valle, Marianne Nymark, Inga Aamot, Kasper Hancke, Per Winge, Kjersti Andresen, Geir Johnsen, Tore Brembu, Atle M. Bones

**Affiliations:** 1 Department of Biology, Norwegian University of Science and Technology, N-7491 Trondheim, Norway; 2 Institute of Biology, University of Southern Denmark, Campusvej 55, DK-5230 Odense M, Denmark; University of Hyderabad, India

## Abstract

Due to the selective attenuation of solar light and the absorption properties of seawater and seawater constituents, free-floating photosynthetic organisms have to cope with rapid and unpredictable changes in both intensity and spectral quality. We have studied the transcriptional, metabolic and photo-physiological responses to light of different spectral quality in the marine diatom *Phaeodactylum tricornutum* through time-series studies of cultures exposed to equal doses of photosynthetically usable radiation of blue, green and red light. The experiments showed that short-term differences in gene expression and profiles are mainly light quality-dependent. Transcription of photosynthesis-associated nuclear genes was activated mainly through a light quality-independent mechanism likely to rely on chloroplast-to-nucleus signaling. In contrast, genes encoding proteins important for photoprotection and PSII repair were highly dependent on a blue light receptor-mediated signal. Changes in energy transfer efficiency by light-harvesting pigments were spectrally dependent; furthermore, a declining trend in photosynthetic efficiency was observed in red light. The combined results suggest that diatoms possess a light quality-dependent ability to activate photoprotection and efficient repair of photodamaged PSII. In spite of approximately equal numbers of PSII-absorbed quanta in blue, green and red light, the spectral quality of light is important for diatom responses to ambient light conditions.

## Introduction

Sunlight is the primary source of energy and a crucial source of information for all photoautotrophs. Marine diatoms, which are responsible for close to 40% of the primary productivity in the world's oceans [1], [2], experience high spatial, temporal and spectral variability in growth irradiance regime due to selective attenuation of solar irradiance in aquatic medium [3]. Longer wavelengths of light are rapidly absorbed by water, with the result that a cell at the surface experiences more red-enriched light than a cell in deeper waters, where green and blue wavebands predominate [4]. In addition to incident solar radiation and time of day, light quality and quantity is also highly affected by the presence of coloured dissolved organic matter (cDOM) and suspended particles [4]. Free-floating diatom cells experience constant vertical displacement in the water column due to tides, waves and currents, and consequently require a high degree of photobiological flexibility. Through a complex system of photoreception and sensory- and metabolic pathways [5], [6], [7], diatoms manage to continuously sense, evaluate and acclimate their photosynthetic apparatus to changes in the intensity of the downwelling irradiance (Ed, Photosynthetically Available Radiation (PAR); 400–700 nm), its spectral quality (E_λ_), its orientation and the day length [8].

The main pigments responsible for harvesting the light-energy that fuel photosynthesis in diatoms include chlorophylls (Chl) *a* and *c* and the carotenoid fucoxanthin (Fuco), bound to fucoxanthin-chlorophyll *a*, *c*-binding antenna proteins (FCPs) [9], [10]. Whereas the chlorophylls absorb light in the blue (400–500 nm) and red (600–700 nm) wavebands of the PAR spectrum, the main accessory pigment Fuco is responsible for the capability of diatoms to absorb light within the green region (500–570 nm). In 2008 and 2009, work by Premvardhan et al. [11], [12] led to the classification of Fuco into two each of high (Fuco_blue_) and low (Fuco_red_) energy absorption and 1–2 of intermediate (Fuco_green_) energy absorption that are allied to their location and function in the LHC proteins of the antenna. Their respective absorption maxima in solvent (MeOH) after extraction from the thylakoids, are located at 445 and 463 nm (Fuco_blue_), 488 and 492 nm (Fuco_green_) and 505 and 510 nm (Fuco_red_). However, in the FCP complexes they absorb in a much wider range of wavelengths (390–580 nm) than in solution (400–520 nm) [12]. This reflects the various conformations and local environments in the LHC proteins, and these differences in absorption and electrostatic properties among the Fuco reflect the important role of the protein environment in fine-tuning the light-harvesting properties of the pigments.

Complementary chromatic adaptation (CCA) is a well-known mechanism in cyanobacteria involving a massive restructuring of the light-harvesting antenna in response to changes in the ambient light quality [13]. This phenomenon allows the microorganism to modify the pigment composition of the antenna to match the wavebands available for photosynthesis. The less flexible FCPs of diatoms prevent them from performing classical CCA. However, recent research has shown that the absorption characteristics of the light harvesting antenna are influenced by light intensity dependent changes in the amount of the three different forms of fucoxanthin (Fuco_blue_, Fuco_green_, Fuco_red_) and the LHC proteins to which they are bound [14]. Low light (LL) acclimated cells have been found to be enriched in Fuco_red_, whereas cultures grown in higher light contain more Fuco_blue_. The exact effect of light quality on the protein-pigment composition of the FCPs has not yet been established.

There are two main mechanisms through which primary producers can sense changes in the ambient growth light [15]. 1) Variations in the rate of photosynthetic electron transport, results in a changed metabolic state of the chloroplast. This change is translated into the up- and down- regulation of nucleus-encoded plastidial proteins through retrograde (organelle-to-nucleus) signaling, which eventually will acclimate the organellar functions to the new light conditions [16]. The reduction/oxidation (redox) state of certain electron transport chain (ETC) components and redox-active soluble compounds have been found to function as signaling parameters involved in adjustment of the photosystem stoichiometry in plants and algae [17], [18], [19], [20], [21]. Several other plastidial signals of different origins (e.g. intermediates of pigment biosynthesis, reactive oxygen species (ROS) and metabolite pool changes) have also been suggested to regulate nuclear gene expression [15], [16], [22]. 2) Photoreceptors constitute the second mechanism sensing irradiance changes in photosynthetic organisms [23]. Different classes of photoreceptors absorb light of various wavelengths depending on the chromophore present in the protein [23], [24]. The absorbed light is converted into biochemical signals that ultimately change the biological activity of cells and organisms [23]. The diatom genomes sequenced to date encode several blue-light receptors [6], that belong to the family of aureochromes found only in photosynthetic Stramenopiles [26], as well as receptors belonging to the widely distributed cryptochromes [27]. Phytochromes, which are red/far-red light receptors, have also been identified in diatoms [6], [25]. Even though light in the green region of the PAR spectrum is the dominating waveband in fjord- and coastal waters [4], no green light photoreceptors have yet been identified in diatoms. Knowledge about the biological function of the different photoreceptors in diatoms is sparse [28]. The signaling cascades and regulatory processes activated by the photoreceptors are basically unknown [28], and none of the downstream components found in plant photoreceptor pathways are present in diatoms [28], [29].

Algal responses to differences in spectral light have been extensively studied. However, most of these studies have not made a clear distinction between the responses due to spectral quality and those due to total irradiance [30], [31], [32], [33], [34], [35], [36], [37]. Pigments absorb light in a highly discriminatory way depending on wavelength; consequently, spectrally dependent responses have to be studied taking the algal absorption spectra into account [38], [39], [40], [41], [42], [43].

To investigate differences caused by light quality alone, we calculated the intensities of blue (BL), green (GL) and red (RL) light providing the same rate of photons for absorption by the algae and thus available for photosynthetic energy transfer, in the marine diatom *Phaeodactylum tricornutum* [44]. We combined time series studies of pigment composition, photosynthetic efficiency and capacity (*in vivo* chlorophyll *a* fluorescence kinetics), and transcriptional regulation through genome-wide transcriptional profiling in order to study the functional connection between molecular response patterns and effects at the metabolic and physiological level. The samples that constituted the base for our previous study on molecular and photosynthetic responses to darkness-white light (WL) transitions [45] were included in selected analyses of the present study. This was done to enable a comparison between acclimation to light of different spectral quality to WL-acclimation.

## Results

*P. tricornutum* cells cultured in continuous white light (CWL) were kept in darkness for 48 h (D48) before being re-exposed to either the initial white light (WL) or to blue light (BL), green light (GL) or red light (RL) at equal Photosynthetically Usable Radiation (PUR). The incubation times were 0.5 h, 6 h and 24 h. Material was harvested from all treatments and time-points. Global gene expression status, cellular pigment concentration, photosynthetic parameters and *in vivo* pigment light energy transfer efficiency (ETE) were examined in the harvested material.

### Transcriptional profiling of nuclear and plastid transcripts and light quality dependency

The differences in molecular acclimation to light of BL, GL and RL compared to WL at corresponding time points were elucidated, focusing on the regulation of genes encoding photosynthesis-related proteins, proteins involved in assembly and repair of photodamaged PSII, ROS scavenging enzymes and photoreceptors. The microarray analysis revealed clear differences in the transcriptional profiles of cultures exposed to the individual treatments, particularly at the 0.5 h time point. Transcripts represented by approximately 43% of the probes on the microarray were significantly differentially regulated when comparing RL-treated cells with cultures exposed to WL for 0.5 h. For GL and BL-treated cells, the corresponding numbers were 22% and 11%, respectively. The differences diminished with light exposure time, but RL proved to be most different from WL also after 6 h and 24 h of treatment. While the amount of regulated transcripts decreased to below 2% when comparing cells exposed to 24 h of BL or GL with WL-treated cells, the number of regulated probes was almost five times higher for RL-treated cultures. Probes representing chloroplast genes do not always produce reliable expression ratios, and these ratios need to be controlled by qRT-PCR [45], [46]. The microarray analyses indicated that photosynthesis-related chloroplast genes, contrary to large amounts of the nuclear genes, responded in much the same way regardless of what wavelengths the cells were exposed to. QRT-PCR of selected chloroplast genes encoding proteins necessary for photosynthesis confirmed these indications (Table S1).

### Gene expression categories of photosynthesis associated nuclear genes

The results of the transcriptional analyses of D48-treated cells and cells re-exposed to WL described in Nymark et al. (2013), suggested that a majority of the photosynthesis associated nuclear genes (PhANGs) and a few photoreceptor genes could be divided into three main categories based on their gene expression profiles. Category 1 included genes encoding light-harvesting complex (LHC) proteins, enzymes necessary for formation of pigments and proteins comprising PSII and other photosynthetic electron transport chain (ETC) components [45]. The gene expression profile representing the category 1 genes was characterized by low transcriptional activity in darkness, modest induction after 0.5 h re-exposure to WL and generally high expression when exposed to WL for longer periods of time. Category 2 described genes encoding proteins suggested to be involved in photoprotection, and were strongly, but transiently induced by the moderate light intensity and peaked after 0.5 h of re-exposure to light. In this study we have also added genes encoding proteins involved in repair and assembly of PSII and a few genes that encode ROS scavenging enzymes to the category 2 group. The expression of genes assigned to the category 3 group peaked after prolonged darkness and showed a gradual reduction towards the initial levels during 24 h of WL re-exposure. The expression of genes belonging to the first two categories will be further commented in this paper.

**Figure 1:**
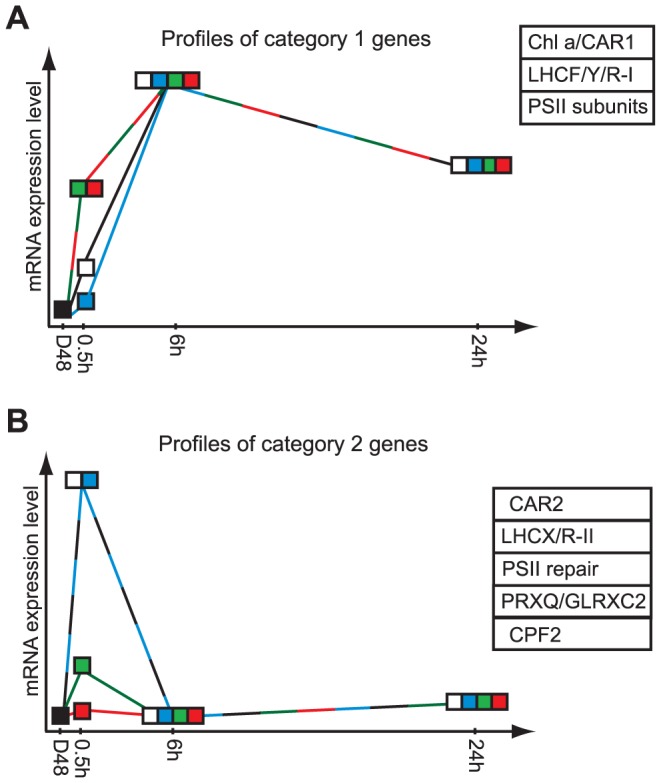
Schematic gene expression profiles representing category 1 and 2 genes. Light quality-dependent gene expression patterns describing the transcript levels after 48 h dark treatment (D48) followed by 0.5 h, 6 h, and 24 h exposure to white light (WL), blue light (BL), green light (GL) or red light (RL) for genes defined as category 1 genes (A) and category 2 genes (B). See text for a detailed description of the different gene expression profiles. Gene groups containing members displaying the different expression profiles are indicated in boxes next to the appropriate figure. The abbreviations used are LHCF: major fucoxanthin Chl *a/c* proteins; LHCR-I: red algal-like proteins (light harvesting); LHCR-II: red algal-like proteins (photoprotective); LHCX: LI818/LHCSR-like proteins; LHCY: deviant light harvesting proteins; Chl *a*: enzymes involved in synthesis of chlorophyll *a*; CAR: enzymes involved in synthesis of carotenoids (CAR1  =  LCYB, ZEP1, ZEP2; CAR2  =  ZDZ, ZEP3); PSII: Photosystem II; PSII repair: proteins involved in PSII repair (PSB27, PSB29, HCF136); PRXQ: Peroxiredoxin Q; GLRXC: Glutaredoxin; CPF2; cryptochrome/photolyase family protein 2.

The exposure of *P. tricornutum* cells to light of different qualities, in addition to WL, supported to a great extent the division of most of the above-mentioned genes into category 1 or 2, by producing light quality-dependent gene expression patterns specific for each of the two categories. The category 1 and 2 gene expression patterns are drawn schematically in Figure 1. BL caused a low to moderate and in some cases no induction of the category 1 genes after 0.5 h of light exposure (Figure 1A). GL in particular, but also RL induced significantly stronger expression of genes in this category than BL, whereas the WL gene responses was intermediate and most often varied between the level found in BL and RL treated cultures. The differences observed after 0.5 h were balanced out with prolonged light exposure time (6 h and 24 h). In contrast to the category 1 genes, expression analyses of several of the category 2 genes revealed that 0.5 h of BL led to a stronger or equal expression as WL, whereas transferring the diatom cultures from darkness to GL or RL led to a much lower or no rise in expression level of these genes at the 0.5 h time-point (Figure 1B).

### Early responses of the Chl a and non-mevalonate pathway genes were light quality-dependent

**Figure 2:**
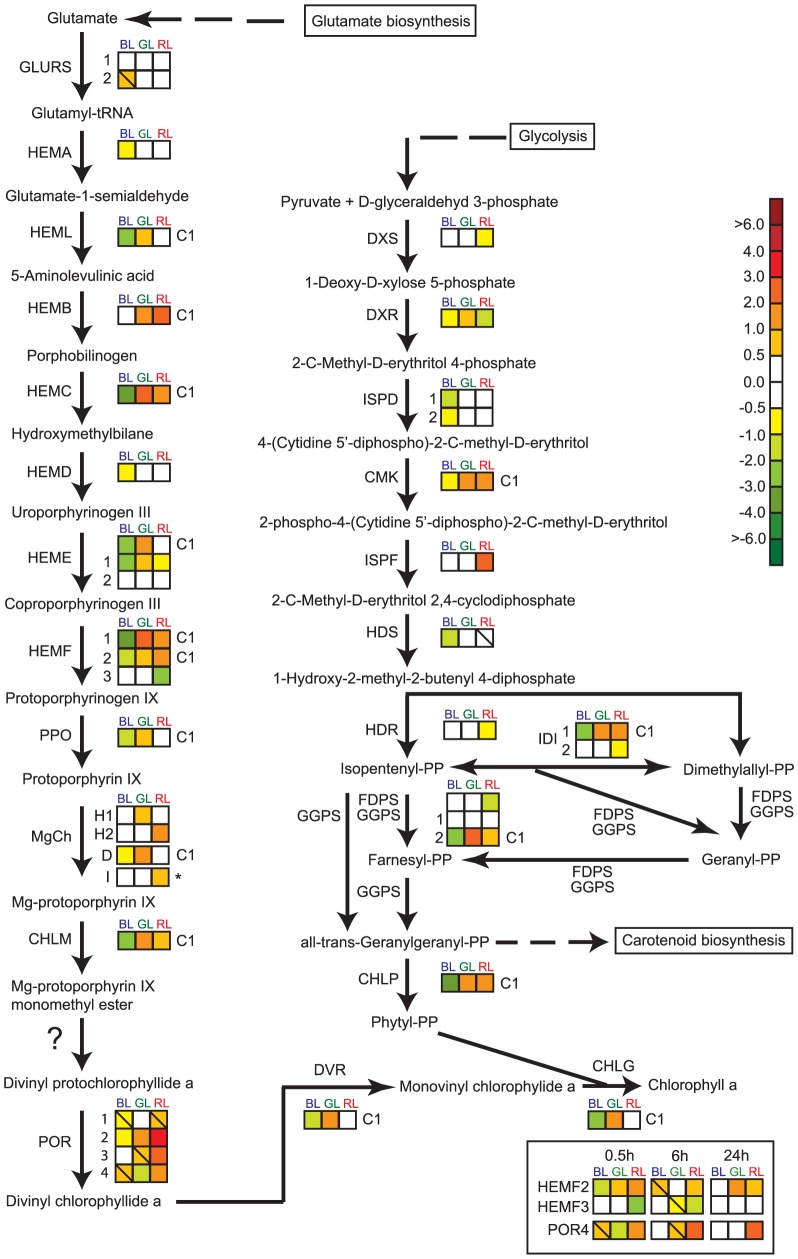
Expression ratios for genes in the hypothesized chlorophyll *a* and non-mevalonate pathways in *P. tricornutum*. Coloured squares indicate the gene expression ratio between blue (BL), green (GL) or red light (RL) exposed cultures compared to white light (WL) treated cultures in the two pathways at the 0.5 h time point. Only *HEMF2*, *HEMF3* and *POR4* were differentially regulated when exposed to prolonged light treatment (6–24 h). The box in the lower right hand corner display the expression ratio for these three genes. Squares with a diagonal line inside indicate genes with an expression ratio greater than +/− 0.5 that are not significantly regulated. The asterisk marking the expression pattern of subunit I of Mg-chelatase (MgCh) indicates that the gene is chloroplast encoded, and that the expression ratios were assessed by qRT-PCR instead of microarray analysis. Genes that display the category 1 expression pattern are marked C1. The scale on the right represents gene expression ratio values, log_2_ transformed. The gene encoding Mg-protoporphyrin IX monomethyl ester cyclase, responsible for converting Mg-protoporphyrin IX monomethyl ester to divinyl protochlorophyllide *a* in higher plants [47], is absent in *P. tricornutum*, and this step is marked with a question mark in the figure. The abbreviations used are: GLURS: glutamyl-tRNA synthetase; HEMA: glutamyl-tRNA reductase; HEML: glutamate-1-semialdehyde 2,1-aminomutase, HEMB: porphobilinogen synthase; HEMC: hydroxymethylbilane synthase; HEMD: uroporphyrinogen-III synthase; HEME: uroporphyrinogen decarboxylase; HEMF: coproporphyrinogen III oxidase; PPO: protoporphyrinogen oxidase; MgCh: magnesium chelatase (comprising of subunits H, D and I); CHLM: Mg-protoporphyrin IX methyl transferase; POR: protochlorophyllide oxidoreductase; DVR: divinyl protochlorophyllide a 8-vinyl reductase; CHLG: chlorophyll synthase; DXS: deoxyxylulose-5-phosphate synthase; DXR: 1-deoxy-D-xylulose 5-phosphate reductoisomerase; ISPD: 2-C-methyl-D-erythritol 4-phosphate cytidylyltransferase; CMK: 4-diphosphocytidyl-2-C-methyl-D-erythritol kinase; ISPF: 2-C-methyl-D-erythritol 2,4-cyclodiphosphate synthase; HDS: 1-hydroxy-2-methyl-2-(E)-butenyl 4-diphosphate synthase; HDR: 4-hydroxy-3-methylbut-2-enyl diphosphate reductase; FDPS: farnesyl diphosphate synthase; GGPS: geranylgeranyl pyrophosphate synthase; IDI: isopentenyl pyrophosphate:dimethylallyl pyrophosphate isomerise; CHLP: geranylgeranyl reductase.

All genes encoding enzymes in the multistep Chl *a* and non-mevalonate pathways leading to the formation of Chl *a* [46], [47], except *GLURS1*, were differentially regulated by light of at least one of the different wavebands after 0.5 h exposure time (Figure 2). After 6–24 h of light exposure, these genes displayed similar high expression levels regardless of light quality. Only three genes encoding HEMF2, HEMF3 and POR4 showed different responses to the light treatments at the two latter time-points as well (Figure 2). The genes in the Chl *a* pathway and the genes involved in the latest steps before formation of phytyl diphosphate previously characterized as category 1 genes [45], displayed a common gene expression profile also as a response to exposure to the different light qualities (Figure 1A, Figure 2). The strongest induction was generally observed in GL, followed by RL, WL and BL.

### Light quality and synthesis of carotenoids

Fuco, the major accessory light harvesting pigment in diatoms, and Diadinoxanthin (Diadino), which can be de-epoxidized to Diatoxanthin (Diato) in high light (HL) as protection against photodamage, are important products of the carotenoid biosynthetic pathway. In spite of recent progress in elucidating this pathway [48], candidate genes for key steps in formation of the end products Fuco and Diadino are still lacking. All carotenoid genes and gene candidates were differentially regulated by light of at least one of the different wavebands after 0.5 h exposure time (Figure S1). Exposure to 24 h of BL, GL or RL balanced out all differences in gene expression compared to WL. The three carotenoid associated genes (*LCYB*, *ZEP1* and *ZEP2*) previously assigned to the category 1 group of genes [45] were found to display the same pattern as the Chl *a* genes, with GL and/or RL exposure leading to the highest initial transcription levels. BL and/or WL were the strongest inducers of expression of the *PDS*, *ZDS*, *ZEP3*, *VDE* and *VDR* genes, supporting the previous grouping of *ZDS* and *ZEP3* with the category 2 genes.

### Green and red lights were the strongest inducers of LHC genes encoding antenna proteins involved in light harvesting

**Figure 3:**
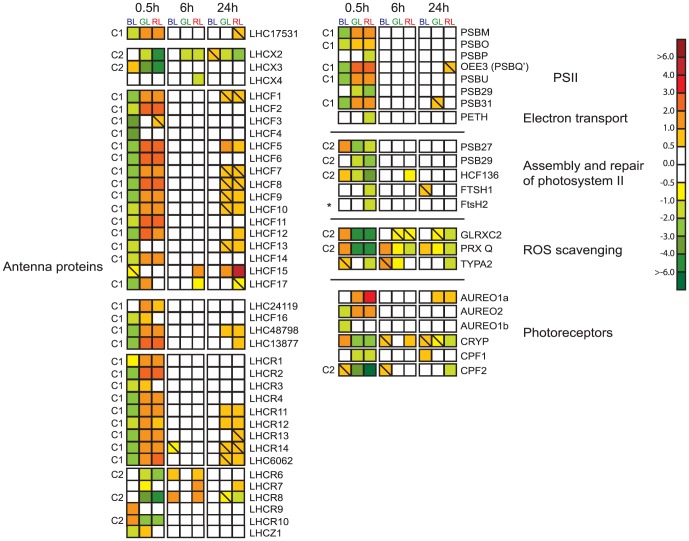
Light quality-dependent expression patterns of genes encoding photoreceptors and proteins associated with photosynthesis, PSII repair and protection against photodamage. The differentially regulated genes encode antenna proteins, components involved in oxidative photosynthesis, assembly and repair of photodamaged PSII, ROS scavenging and photoreceptors. The gene expression ratios are produced as a response to blue (BL), green (GL) or red light (RL) exposure for 0.5 h, 6 h or 24 h. White light (WL) at corresponding time-points were control conditions. The colour code indicates expression values, and squares with a diagonal line inside indicate genes with an expression ratio (log_2_ transformed) greater than +/− 0.5 that are not significantly regulated. Genes where at least one of the two probes used for calculation of the expression ratios were significantly regulated by>2-fold at least at one time point during the experimental period were included in the figure. The *FtsH2* gene is chloroplast-encoded and marked with an asterisk. The expression ratios for *FtsH2* was assessed by qRT-PCR instead of microarray analyses. The nucleus-encoded *LHC48798* gene was not represented on the microarray, and was therefore also assessed by qRT-PCR analyses. Genes that display the category 1 and 2 expression patterns are marked C1 and C2 respectively. The scale on the right represents gene expression ratio values, log_2_ transformed. The abbreviations used are LHCF: major fucoxanthin Chl *a/c* proteins; LHCR: red algal-like proteins; LHCX: LI818-like proteins; LHCZ: unknown function antenna protein, LHC#: LHCY proteins, previously unclassified light harvesting proteins, numbers refer to protein ID in JGI; PSB: PSII proteins; PETH: Ferredoxin-NADP reductase; HCF: high Chl fluorescence; FTSH: Filamentation temperature sensitive H; GLRXC: glutaredoxin; PRX: peroxiredoxin; TYPA: tyrosine phosphorylation protein A; AUREO: aureochrome; CRYP: cryptochrome-like protein; CPF: cryptochrome/photolyase family protein.

Phylogenetic analyses including all *P. tricornutum* antenna proteins belonging to the light harvesting complex (LHC) superfamily suggested the existence of four main LHC groups: the major fucoxanthin Chl *a*/*c* proteins (LHCFs), the red algal-like LHCRs, the stress-responsive LI818/LHCSR-like LHCXs and the newly identified LHCY group, which mainly consists of previously unclassified LHCs [45]. The LHCR group was further divided into to separate subgroups, LHCRI and LHCRII. The response of the *P. tricornutum* LHC genes to the BL, GL and RL treatments compared to WL is presented in Figure 3. All LHC genes characterized as category 1 genes [45], encoding proteins belonging to the LHCY group, the LHCF group (except LHCF15 and LHC42519), the LHCRI group including the LHC6062 protein and the deviant LHC17531, exhibited the light quality-dependent expression profile common for the category 1 genes (Figure 1A). GL and RL resulted in the strongest gene induction, while WL and BL were weaker inducers of these genes. After 24 h of light exposure, approximately half of these genes exhibited somewhat higher expression levels in GL and RL than in BL and WL (Figure 3).

While the expression level of most genes encoding components of the light-harvesting antenna was relatively similar between the different wavelengths after 6–24 h of light exposure, the response of *LHCF15* to RL after D48 was unique. *LHCF15* was highly expressed in darkness [45], and reached even higher and similar expression levels after exposure to 0.5 h light of the different qualities. The amount of gene transcripts peaked at the 0.5 h time point and decreased at a similar rate during the next 24 h when exposed to WL, BL or GL. Exposure to RL, on the other hand, caused practically no decrease in *LHCF15* transcripts and the expression remained at high levels throughout the experiment (Figure 3).

Several of the members of the LHCRII group (*LHCR6-8*, *LHCR10*) and the LHCX group (*LHCX2-3*) have been characterized as category 2 genes [45]. These genes are believed to be involved in photoprotection and are induced by HL [45], [46], [49], [50]. BL and WL provoked a strong induction of these genes, while GL and RL only caused a weak or no gene induction (Figure 1B, Figure 3). The *LHCX1* gene, encoding a LHC protein involved in non-photochemical quenching (NPQ) [50], was expressed at high levels at all times, regardless of light quality, and was not found to be significantly regulated at any time point.

### Expression of the chloroplast-localized genes encoding the multi-subunit complexes PSII, PSI, the cytochrome b6f complex and F-ATPase was light quality-independent

Oxygen evolving photosynthesis takes place in the chloroplast and is catalyzed by the four multi-subunit complexes PSII, PSI, the cytochrome *b*6*f* complex and F-ATPase embedded in the thylakoid membrane [51]. Chloroplast-localized genes encode most of the building blocks of these complexes, with the PSI complex containing only chloroplast-encoded subunits [6], [52]. Microarray analyses indicated that the chloroplast gene response was mainly independent of light quality. The expression of six chloroplast genes (*PsaA, PsaE, PsbA, PsbV, PetB, AtpB*) encoding subunits of each of the above-mentioned complexes were determined by qRT-PCR. None of these genes displayed significant regulation (more than two-fold) at any time-point during the experiment, and are therefore not included in Figure 3. The expression ratios obtained by qRT-PCR supported the light quality-independent response of the photosynthesis-related chloroplast-encoded genes indicated by the microarray analyses. The chloroplast-encoded PsbV and the nucleus-encoded PSBO, OEE3 (PSBQ'), and PSBU comprises the diatom oxygen-evolving complex (OEC) in PSII. The rate of *PsbV* gene transcription was unaffected by light of different quality. In contrast, the nucleus-localized OEC genes displayed the same response to light of different wavebands as described for other photosynthesis-related category 1 genes (Figure 1A, Figure 3). The expression pattern described in Figure 1A was also found to be valid for the category 1 genes *PSBM* and *PSB31* [45], which encode other PSII subunits. The *PETH* gene encoding ferredoxin-NADP reductase, and other nucleus-localized genes encoding subunits of PSII, the cytochrome *b*6*f* complex and F-ATPase, showed limited or no differential responses to treatments with light of different quality.

### Genes involved in repair of photodamaged PSII were strongly induced in blue light, intermediately expressed in green light and weakest induced in red light

Photodamaged PSII is continuously repaired by partial disassembly of the PSII complex followed by D1 protein degradation and re-synthesis, and finally reassembly of the PSII complex [53]. The expression of several genes encoding proteins known from other organisms to be involved in PSII repair [53], [54] were found to be light quality-dependent in this study (Figure 3). BL, and to a lesser extent WL, caused a transient rise in the expression of the *PSB27* (only induced by BL), *PSB29* and *HCF136*, which encode proteins associated with repair of PSII after 0.5 h light exposure. GL caused a more modest induction of these genes than WL, whereas RL exposure was not able to boost the expression of these genes above D48 levels. Longer light exposure times caused similar and more moderate expression levels of these genes fitting the category 2 expression profile (Figure 1B). The PSII repair genes generally showed higher basal expression levels at the D48, 6 h and 24 h time points than the photoprotective LHC genes also defined as category 2 genes. FTSH proteases are important for degradation of photodamaged D1 protein [55], [56]. The nucleus-localized *FTSH1* gene and the chloroplast-localized *FtsH2* gene both displayed significantly lower expression levels as a response to 0.5 h of RL, compared with the other wavebands.

### ROS scavenging system genes were boosted in blue light while remaining at dark-level in green and red light

Photosynthetic organisms have developed efficient scavenging systems to handle ROS generated by the light reactions of photosynthesis [57], [58], [59]. The genes encoding peroxiredoxin Q (PRXQ), glutaredoxin 2 (GLRXC2) and two members of the TypA/BipA GTPase family (TYPA1, TYPA2) displayed the strongest and most consistent up-regulation among the ROS scavenging system genes after a LL-HL shift [46]. Based on its co-expression with *PRXQ* during HL, GLRXC2 has been suggested to be the redox enzyme responsible for the reactivation of PRXQ [46]. *PRXQ* and *GLRXC2* were both strongly induced by 0.5 h of BL, whereas the expression level remained close to the initial low D48 levels in GL and RL-exposed cells (Figure 3). Prolonged exposure time generally led to low (although not identical; Figure 3) expression levels, similar to the category 2 expression profile (Figure 1B). *TYPA1* was only modestly affected by exposure to light of different quality, and was not included in Figure 3 since this gene was never regulated more than two-fold at any time point or by any light treatment. *TYPA2* was not transcribed in D48 cells. Light exposure of all qualities induced the transcription of this gene, but RL provoked lower transcript levels than the other wavebands at the 0.5 h time point (Figure 3). In RL, *TYPA2* was expressed at a constant and moderate level throughout the length of the experiment, whereas the expression levels in WL, BL and GL peaked after 0.5 h of light exposure.

### Light quality and expression of photoreceptor genes

Six genes encoding blue light receptors belonging to two different families (aureochromes and cryptochromes) were found to be differentially regulated by light of different quality (Figure 3). As for the majority of other genes described in this study, the blue light photoreceptor genes displayed the most variable responses to different quality light at the 0.5 h time-point. Longer light exposure times led to similar gene expression levels regardless of light quality. The aureochromes function as BL-activated transcription factors [60], [61], but might also function as transcriptional repressors [62]. *AUREO1a* and *AUREO2* have higher expression in D48-treated cells than in CWL . Re-exposure to 0.5 h of WL after D48-treatment caused a significant decrease in gene transcription. Exposure to BL for 0.5 h had a similar effect on transcription as WL, whereas GL and RL exposure led to a more modest decrease in gene expression (Figure 3). Coesel et al. [63] showed that the *P. tricornutum* cryptochrome/photolyase family protein CPF1 had both DNA repair and transcription regulation activity. D48-treatment led to a strong increase of *CPF1* transcription level [45]. In contrast to the observed decline in *AUREO1a* and *AUREO2* gene transcripts in response to 0.5 h exposure to WL and BL, *CPF1* transcription levels stayed at the high D48 level when subjected to the same treatment. Exposure to 0.5 h of GL and RL caused a decrease in the amount of *CPF1* transcripts. When exposed to longer periods of light of all different qualities, the amount of *CPF1* gene transcripts declined to similar and very modest expression levels. *CRYP* (*CPD2* in [28], *CRYL* in [45], [46]) encodes a novel cryptochrome that influences the LHC protein levels [64]. The expression of *CRYL* peaked after D48 treatment and responded in a highly similar way to light of different quality as *CPF1*; however, WL also caused a decrease in transcription levels after 0.5 h light exposure, although to a more modest degree than GL and RL. A member of the cryptochrome-DASH family encoded by *CPF2* has been designated to the category 2 group [45]. The *CPF2* expression profile produced in response to light of different quality displays the same pattern as found for other category 2 genes (Figure 1B). *CPF2* was not expressed in D48-treated cells, and the expression was low to moderate when exposed to light of different quality for longer periods of time. However, at the 0.5 h time point a strong induction of transcription was observed when exposing the cells to BL and to a lesser degree to WL. GL proved to be a very weak inducer of *CPF2* transcription, whereas no induction of *CPF2* was detected by exposing the cells to RL for 0.5 h.

### The strong induction of genes involved in photoprotection in blue light was unaffected by loss of the photosynthetic signal

**Figure 4:**
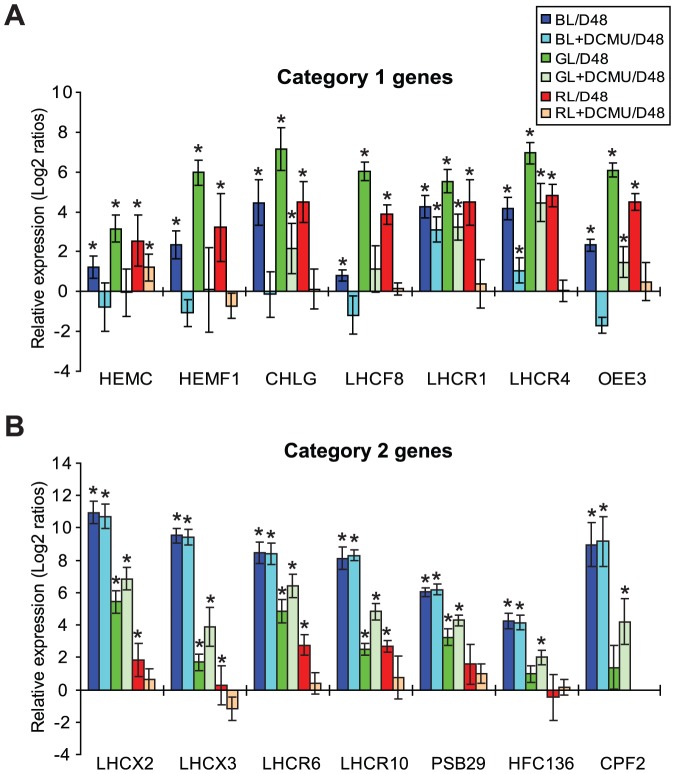
Transcriptional responses after exposure of *P. tricornutum* cells to light of different quality with or without the addition of the photosynthetic inhibitor DCMU. Bars represent relative gene expression values (log_2_ transformed) in blue (BL), green (GL) and red light (RL)-treated cultures with or without the addition of DCMU prior to light exposure, compared to 48 h dark-treated cells (D48) for a subset of category 1 (A) and category 2 (B) genes. *RPS5* was used as a reference gene. *CPF2* is not transcribed in D48-treated cells or in RL-exposed cells (concluded from the microarray analyses). Bars representing *CPF2* gene expression in RL are therefore omitted from the figure. Relative *CPF2* expression values are calculated against the low background levels caused by minute amounts of genomic DNA amplified during the qRT-PCR reaction. Bars marked with an asterisk indicate that gene expression is significantly different in light-treated cultures compared to D48 levels (log_2_ ratios> +/−1.5 and p<0.05). Error bars show the 95% confidence intervals. The abbreviations used are: DCMU: 3-(3,4-dichlorophenyl)-1,1-dimethylurea; HEMC: hydroxymethylbilane synthase; HEMF1: coproporphyrinogen III oxidase 1; CHLG: chlorophyll synthase; LHCF: major fucoxanthin Chl *a/c* proteins; LHCR: red algal-like proteins; LHCX: LI818-like proteins; OEE3: Oxygen-Evolving Enhancer Protein 3; Psb29: photosystem II biogenesis protein Psp29; HCF136: high Chl fluorescence 136 (photosystem II stability/assembly factor); CPF2: cryptochrome/photolyase family protein 2; RPS5: 30S ribosomal protein S5.

To discriminate between gene induction caused by photoreceptor mediated signals and retrograde signaling from the chloroplast, the effect of DCMU addition prior to 0.5 h BL, GL or RL exposure was examined in a subset of category 1 genes (*HEMC*, *HEMF1*, *CHLG*, *LHCF8*, *LHCR1*, *LHCR4*, *OEE3*) and category 2 genes (*LHCX2*, *LHCX3*, *LHCR6*, *LHCR10*, *HCF136*, *PSB29*, *CPF2*). DCMU binds to the Q_B_ pocket of the D1 protein of PSII and blocks the transfer of electrons from PSII to the plastoquinone (PQ) pool, thereby inhibiting linear photosynthetic electron transport (PET) [8]. The experimental setup of the inhibitor study and subsequent qRT-PCR analyses enabled both a comparison between gene expression levels in cultures exposed to 0.5 h of light of different quality with or without the addition of DCMU (Table S2), and a comparison of gene expression levels in the mentioned cultures against D48-treated cells (Figure 4).

Blocking of linear PET caused a considerably weaker induction of the selected category 1 genes compared to the gene induction in the corresponding light-treated cultures without the photosynthetic inhibitor (Figure 4A, Table S2). The comparison of gene expression in DCMU+light-treated samples to D48-treated samples revealed that the expression level of several of the category 1 genes did not rise above D48-levels during the 0.5 h DCMU+light exposure time, especially in the cultures treated with DCMU+BL or DCMU+RL (Figure 4A). The transcriptional analyses of four of the category 1 genes (*HEMC*, *HEMF1*, *LHCF8* and *OEE3*) indicated that the expression level in DCMU-treated BL cultures might even be lower than in the D48 treated cultures. In contrast, GL was capable of inducing transcription of several category 1 genes, also when DCMU were added to the cultures prior to light exposure (Figure 4A).

Removing the possibility to perform linear PET had a light quality-dependent effect on the induction of the category 2 genes (Figure 4B). Addition of DCMU prior to BL treatment had practically no impact on the strong induction of the category 2 genes. These genes reached equally high expression levels in BL-exposed cultures both with and without DCMU (Figure 4B, Table S2). Cultures treated with DCMU+GL showed a slight increase in expression of several of the category 2 genes compared to the GL samples (Figure 4B, Table S2), whereas addition of DCMU prior to RL exposure caused the transcript level of these genes to remain at D48-levels (Figure 4B).

### Cellular pigment concentrations

**Figure 5:**
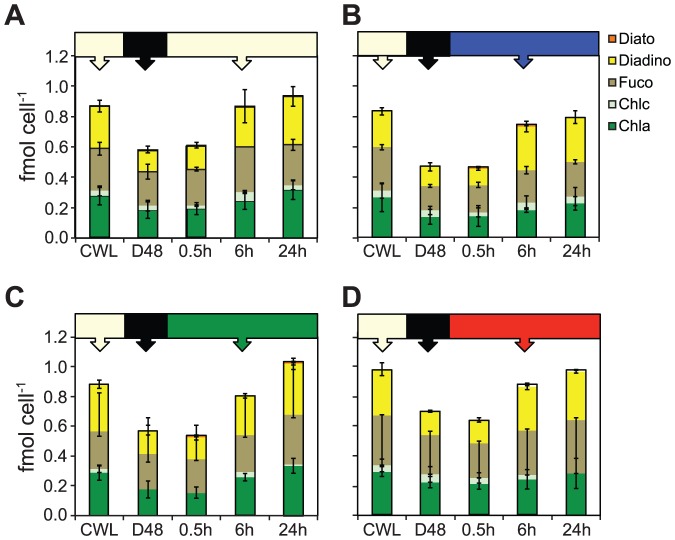
Changes in cellular pigment concentrations during blue, green and red light treatments. All cultures were grown in continuous white light (CWL) of 100 µmol m^−2^ s^−1^ left dark for 48 hours (D48) and subsequently exposed to equal PUR of the (A) initial white, (B) blue, (C) green and (D) red light. The presented data are the mean of three biological replicates and the averages of three parallel HPLC samples. Values are presented with ±SD bars.

The total cellular concentrations of all detected pigments in CWL, D48 and subsequent 0.5–24 h of WL, BL, GL and RL samples are presented as femtomol cell^−1^ in Figure 5. Chls *a* and *c (c_1_+c_2_)*, Fuco and Diadino dominated the cell pigment pool in all samples analysed. Total cellular pigment concentrations decreased 20–40% from the CWL treatment to the D48 treatment, as a consequence of pigment dilution by cell cycle completion in the dark. At 0.5 h after re-exposure to light, few changes had taken place, while pigment concentrations increased considerably with increasing exposure time. In WL, BL and RL-treated cultures, the total cellular pigment concentrations ended up close to their respective CWL levels, while it was significantly different (p<0.05) only in the 24 h GL-treated cells compared to its initial CWL concentration. Furthermore, the total cellular pigment concentration in BL, GL and RL were similar to WL levels after 24 h of light exposure. In the case of cellular Chl *c*, unequal proportions in random samples were mixed with small concentrations of Chl *a,* and could not be separated by the HPLC method. The Chl *c* data are consequently not discussed further.

The Chl *a* fraction to the total cellular pigment concentration remained relatively constant during the course of the WL, BL, GL and RL treatments, as shown in Figure S2. The fraction of Fuco to the total cellular pigment concentration was higher in the D48 samples compared to CWL. It remained high after 0.5 h in all four spectral light treatments, but decreased after 6 h and 24 h of light re-exposure, independent of light quality. The cellular fraction of Diadino to the total cell pigment concentration displayed the inverse pattern of Fuco. It decreased in the D48 samples, remained quite unchanged after 0.5 h and gradually increased after 6 and 24 h of light-re-exposure, again regardless of light quality. Small amounts of Diato were detected at varying time-points in BL (0.5 h and 6 h), GL (0.5 h and 24 h) and RL (6 h and 24 h)-exposed cells.

### Light energy transfer efficiency in the blue, green and red PAR waveband was growth light-dependent

**Figure 6:**
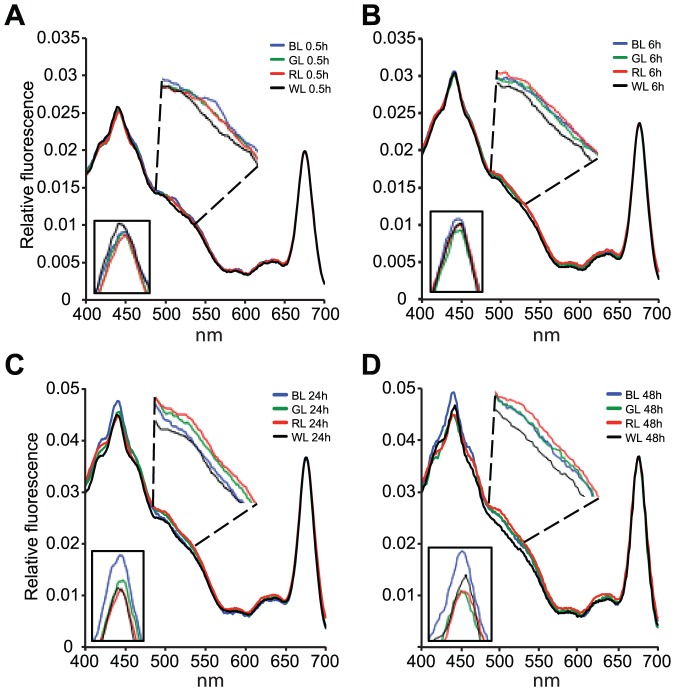
Spectral differences in light energy transfer efficiency (ETE) during blue, green and red light treatments. *In vivo* fluorescence excitation spectra of *P. tricornutum* cultures after A) 0.5 h, B) 6 h, C) 24 h and D) 48 h of blue (BL), green (GL), red (RL) and white (WL) growth light. Framed inset in A-D represents the enlarged part of the spectra within the 430–440 nm range (blue light), which includes the emission maximum of Chl *a* at 440 nm and the region of maximum absorption by Fuco_blue_. Enlarged section of the spectra within the 480–520 nm (blue-green) range is presented within the dashed lines in A-C, and corresponds with the combined maximum absorption range of Fuco_green_ and Fuco_red_.

**Figure 7:**
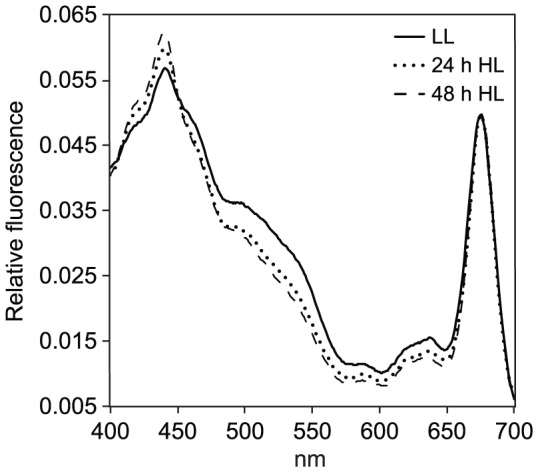
Time series of changes in light energy transfer efficiency (ETE) in *P. tricornutum* during low white light (35 µmol m^−2^ s^−1^) and subsequent exposure to 24 and 48 h of high white light (500 µmol m^−2^ s^−1^). The 24 and 48 h HL spectra were scaled to the LL Chl *a*-fluorescence maximum at 675 nm for comparison.

The fluorescence excitation spectrum of an alga at ambient temperature results from the re-emission of light reaching Chl *a* of PSII (Chl *a* fluorescence) [65], [66], [67]. The emitted light has a longer wavelength than the absorbed light, and has two peaks in intensity at ∼680 [67], [68] and 730 nm [69]. For a fixed wavelength of emission, the intensity of the fluorescence varies with the wavelength of incident light. This wavelength dependence is caused by the differential absorption of light by the pigment–protein complexes associated with PSII [70]. Photoprotective pigments are not included by this technique, as they absorb light but do not transmit the energy to any of the photosystems and hence do not contribute to the fluorescence [71], [72]. Fluorescence originating from PSI is negligibly small [67]. The *in vivo* fluorescence excitation spectrum of an alga grown under a given light regime therefore indicates the relative light energy transfer efficiency (ETE) of the different light-harvesting pigments to the PSII reaction centre Chl *a* within the PAR region (400–700 nm). If an alga is capable of some degree of CCA, light ETE from light-harvesting pigments to the PSII reaction centre would therefore increase with incubation time during growth at a given light colour. The BL, GL, RL and WL samples were analysed at 0.5, 6, 24 and 48 h time-points in order to investigate whether such an increase in ETE could be detected, and the BL, GL and RL spectra from each time-point were scaled to the WL Chl *a*-fluorescence maximum at 675 nm (Figure 6) in order to compare ETE between 400 and 700 nm from Chls *a* and *c* and Fuco to the PSII reaction centre Chl *a*. No differences in ETE between the four spectral light treatments could be detected after 0.5 and 6 h of light re-exposure (Figure 6A and 6B). After 24 h however, a weak tendency appeared, where the RL and GL-grown cells exhibited a slightly lower peak around the *in vivo* excitation maximum of Chl *a* (440 nm) compared to BL (Figure 6C). In contrast, RL and GL cells generally displayed a slightly enhanced ETE in the 480–650 nm range over BL cells at the 24 h time-point. The tendency was even more evident after 48 h (Figure 6D). The ETE of the 48 h RL and GL cells in the 410–480 nm range decreased a total of ∼10% at the *in vivo* excitation maximum of Chl *a* (∼440 nm) compared to BL. In the 480–650 nm range, on the other hand, ETE of the RL and GL cells were dominating. Interestingly, RL-grown cells displayed a higher ETE after 24 and 48 h than GL-grown cells in the entire 480 to 650 range, which also includes the green waveband of the PAR spectrum. The WL cells exhibited the lowest ETE of the light treatments in the entire range at all time-points, except from a small range around the *in vivo* excitation maximum of Chl *a* (∼440 nm). A comparison between the colour-specific ETE responses and the corresponding responses to a LL-HL shift revealed identical patterns in the 24–48 h LL and RL samples, and in the 24–48 h HL and BL samples (Figures 6C and 6D, Figure 7).

### Photosynthetic efficiency remained light quality-independent during the first 24 h, after which it declined in red light

**Figure 8:**
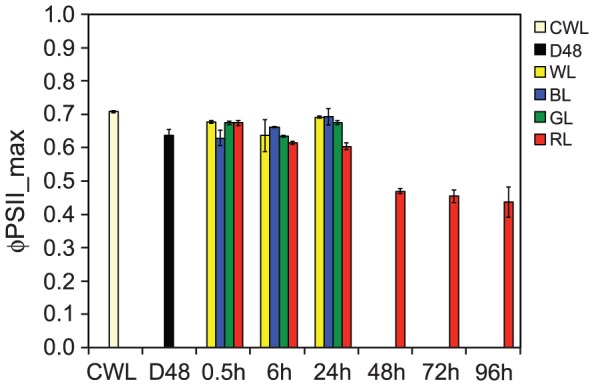
Maximum quantum yield of charge separation in photosystem II (Φ_PSII_max_) of *P. tricornutum* cells as a response to blue, green and red light treatments. All cultures were grown at continuous white light (CWL), dark-treated for 48 h (D48) and subsequently exposed to 0.5, 6 and 24 h of equal PUR doses of white (WL), blue (BL), green (GL) and red (RL) light. Additional measurements after 48, 72 and 96 h were included for the RL treatment. Φ_PSII_max_ was measured directly after a 3 min dark acclimation period and is a measure of the maximum efficiency of photosynthetic electron yield per mol photons absorbed in PSII. All values are presented with ±SD bars (n = 3).

The maximum quantum yield of charge separation in PSII (Φ_PSII_max_) offers a good measure of the photosynthetic efficiency. Φ_PSII_max_ indicated that the differences in photosynthetic efficiency in WL, BL, GL and RL-treated *P. tricornutum* cells were small during the first 24 h of light exposure after D48 treatment (Figure 8). Light re-exposure (0.5 h) after D48 treatment initiated equal amounts of electrons generated to photons absorbed in PSII in WL, GL and RL. All three treatments resulted in approximately the same Φ_PSII_max_ as the initial CWL cells already after this short period of time. Φ_PSII_max_ of the WL and GL-treated cultures stayed at the CWL level for the rest of the experiment. The Φ_PSII_max_ of the BL culture remained at the level of the D48 cells at 0.5 h, but exhibited a gradual increase, reaching CWL levels at the 24 h time-point. In contrast, Φ_PSII_max_ of the RL culture gradually decreased from 0.5 to 24 h, ending up lower than the other light treatments. To establish whether this negative trend would continue, measurements were performed after 48, 72 and 96 h RL. The results revealed that Φ_PSII_max_ continued to decrease throughout the whole period of RL exposure, reaching a level at the 96 h time-point that was ∼35% lower compared with the 0.5 h level (Figure 8).

### High intensity red light caused the photosynthetic efficiency to drop

The declining trend of the Φ_PSII_max_ in the RL samples, in spite of the moderate intensity, prompted us to investigate whether the decreasing photo-physiological trend of the RL-treated cells might be caused by insufficient protection and/or PSII repair activity during RL. Additional samples were exposed to 730 µmol m^−2^ s^−1^ of RL (RL_max_), which was the maximum possible intensity of the red diode light. Harvesting of material for a microarray screen (one sample), qRT-PCR and pigment analysis were performed after RL_max_ exposure for 0.5 h. *In vivo* absorption, fluorescence excitation and variable *in vivo* Chl *a* fluorescence were measured at six additional RL_max_ incubation time points (3 h, 6 h, 24 h, 48 h, 72 h and 96 h).

**Figure 9:**
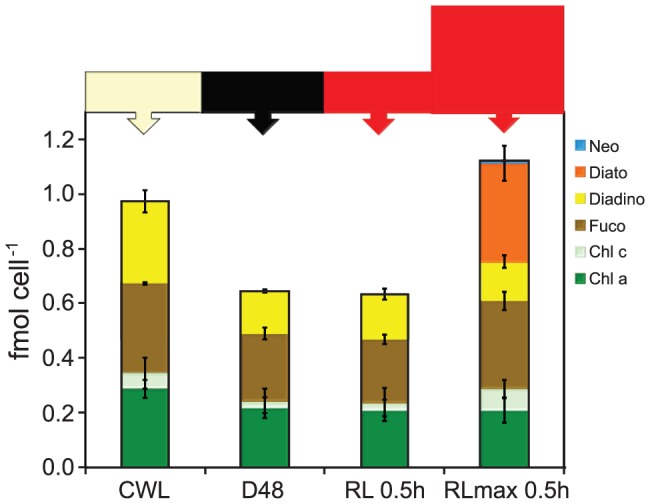
Cellular pigment concentrations from the CWL, D48, 0.5 h RL and 0.5 h RL_max_ treatments. All individual cultures were grown in continuous white light (CWL) of 100 µmol m^−2^ s^−1^, left dark for 48 hours (D48) and re-exposed to 0.5 h of either 230 or 730 µmol m^−2^ s^−1^ of RL. The presented data are the mean of three biological replicates and the averages of three parallel HPLC samples. Values are presented with ±SD bars (n = 3).

The microarray screen indicated that the increased RL intensity (RL_max_) was not able to elevate the expression of the category 2 genes involved in photoprotection and PSII repair any further (data not shown). qRT-PCR analyses of a subset of these genes (*LHCX2, LHCX3, LHCR6, LHCR10, PSB29, HFC136*) confirmed the results indicated by the microarray (Table S3). The pigment data revealed that 0.5 h of RL_max_ caused equally high cellular concentrations of Fuco and Diato, in addition to a small concentration of neoxanthin (Neo) that appeared for the first time in the dataset (Figure 9). In contrast, Chl *a* and Diadino concentrations remained unchanged compared to the D48 cells. As a consequence of the very high Diato concentration in the 0.5 h RL_max_ cells (Figure 9), the total cellular pigment concentration was almost doubled compared to the D48 treatment and the 0.5 h RL cells.

**Figure 10:**
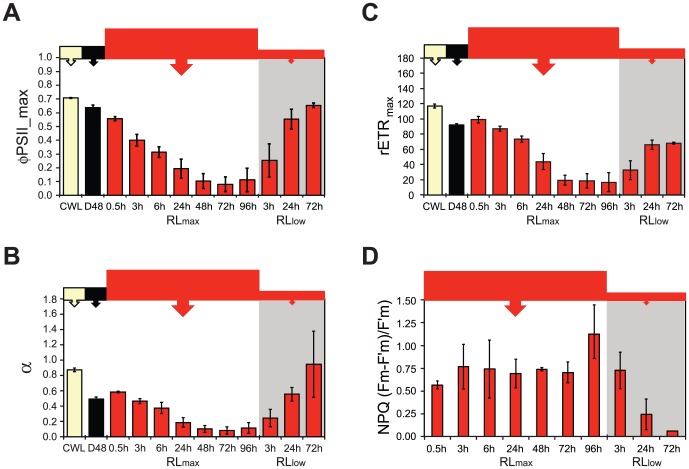
Photo-physiological responses to 0.5–96 h of RL_max_ (730 µmol m^−2^s^−1^) and subsequent 3 to 72 h of recovery during RL_low_ (80 µmol m^−2^s^−1^) by *in vivo* Chl *a* fluorescence kinetics (PAM). A) Φ_PSII_max_, B) α, C) rETR_max_ and D) Non-photochemical quenching (NPQ). All cultures were grown at continuous white light (CWL), dark-treated for 48 h (D48), re-exposed to 0.5 to 96 h of 730 µmol m^−2^ s^−1^ (RL_max_) and subsequently to 80 µmol m^−2^ s^−1^ (RL_low_; gray area to the right) during 3 to 72 h of recovery. NPQ values were calculated according to Stern-Volmer ((Fm-F'm)/F'm). NPQ of the CWL and dark cultures are not included, since diatoxanthin required for NPQ was absent in these samples. All values are presented with ±SD bars (n = 3).

The Φ_PSII_max_ decreased gradually after RL_max_ exposure of the D48 cells until 72 h, at which it was barely detectable (<0.1, Figure 10A). The slight increase in Φ_PSII_max_ at the 96 h time point exhibited high standard deviation, which might indicate a low-level stabilization rather than an actual increase. Subsequent exposure of the 96 h RL_max_ cells to 72 h of 80 µmol m^−2^ s^−1^ red light (RL_low_) resulted in almost 100% recovery of Φ_PSII_max_ compared to the CWL cells (Figure 10A). The α (the slope of the photosynthesis versus irradiance curve, Figure 10B) and rETR_max_ (defined as the photosynthetic capacity, Figure 10C) exhibited the exact same pattern of decline as Φ_PSII_max_, with the exception of a small increase at the 0.5 h time point compared to the D48 cells. The recovery of α was complete after 72 hours, while rETR_max_ only reached approximately half the value of the initial CWL cells during the same period.

The calculated NPQ was relatively high during the entire RL_max_ experiment. Values fluctuated between 0.5 and 0.8 during the 0.5–72 h period (Figure 10D), followed by a sudden increase at the 96 h time-point. Upon transfer to RL_low_, NPQ almost ceased within 72 h. The standard deviation was high in all but two measurements (0.5 h and 48 h).

## Discussion

The purpose of this study was to investigate the response to light of different quality (BL, GL or RL) compared to WL in *P. tricornutum*. By exposing algal cultures synchronized by prolonged dark treatment to equal PUR instead of equal irradiances, we aimed to reduce differences caused by factors other than the quality of light itself. Several observations support that the attempt to provide an approximately equal amount of PUR to be absorbed in PSII has been successful: 1) Lack of differentially regulated photosynthesis-related chloroplast genes, 2) high similarity in expression of nuclear genes, independent of light quality, after prolonged light exposure (6–24 h), 3) the general and light quality-independent trend in variation of pigment concentrations and ratios, and 4) the similar growth rate measured in all cultures during the length of this experiment.

### Light quality-dependent short-term differences in gene expression

Several of the PhANGs and a few genes encoding putative photoreceptors were divided into different categories in Nymark et al. [45] based on common responses to changes in light conditions and exposure times. This categorization is supported for most of these genes by the short-term light quality-specific gene expression patterns produced by exposure to BL, GL and RL. In general, 0.5 h of GL and/or RL exposure function as stronger inducers of the category 1 genes than WL and BL (Figure 1A, Figure 2–3) at the light intensities used in this study. The category 1 genes encode proteins associated with light harvesting and photosynthetic electron transport. After 6 and 24 h exposure, nearly equal and high expression levels were found for most of these genes in all treatments, indicating that the long-term regulation of the category 1 genes is practically independent of light quality (Figure 1A, Figure 2–3). In a study by Schellenberger Costa and co-workers, chloroplast membrane proteins were isolated from cells acclimated to low BL and low RL for one week [42]. Even though their experimental setup differed from ours, both in acclimation status of the cells analysed (acclimated to BL/RL vs. dark to BL/RL shift), light duration (1 week vs. 24 h) and light regime (day/night cycles vs. continuous light), their findings largely support a long-term light quality-independent regulation of proteins encoded by the category 1 genes. With a few exceptions, such as the enrichment of CHLD (Mg-chelatase, subunit D; only identified in BL), LHCF11, LHCR1 and ZEP1 in BL, and LHCF2 and POR1 in RL, similar levels of the category 1 gene products were detected in BL and RL [42].

The category 2 genes are strongly, but transiently induced by 0.5 h of BL and WL exposure following prolonged exposure to darkness (Figure 1B, Figure 3). Several of the category 2 genes that might function in photoprotection (*LHCR6-8*, *LHCR10*, *LHCX2-3*, *PRXQ*, *GLRXC2*) were expressed at elevated levels for hours to days after transfer of LL-acclimated *P. tricornutum* cells to HL, whereas an increased expression of genes encoding PSII repair proteins (Psb27, Psb29, HFC136) were almost exclusively detected after 0.5 h of HL exposure [46]. Transferring the diatom cultures from darkness to GL or RL leads to a much lower or in some cases no rise in expression level of the category 2 genes. None of the proteins encoded by the category 2 genes were detected in either BL or RL in the study by Schellenberger Costa et al. [42]. Non-detectable levels of the category 2 gene products connected to photoprotection can be explained by the LL intensities the cells were acclimated to in the study.

However, LHCX1 and FTSH1-2, which are connected to important photoprotective processes like NPQ and PSII repair respectively, were detected both at the transcriptional level (in our study) and at the protein level in both BL and RL [42]. The amount of LHCX1 and FTSH2 proteins were slightly elevated in cells acclimated to BL compared to RL [42], whereas no significant difference was seen at the transcriptional level after 24 h in our study. BL was found to be essential for HL acclimation and photoprotection in *P. tricornutum*, and the increased LHCX1 protein level in BL compared to RL was suggested to be responsible for the increased photoprotective capacity in BL [42]. In addition to *LHCX1*, *LHCF15* was among the few *LHC* genes that could not be assigned to either of the two gene categories. LHCF15 protein expression is suggested to be RL-specific [42], but even though our data also show a much higher gene expression of *LHCF15* in RL compared to WL, BL and GL after 24 h of light exposure, we also detected similar high transcripts levels after prolonged darkness. Seen together, these results indicate that the high expression of *LHCF15* in RL is not induced by RL, but rather by a lack of light of shorter wavelengths.

### Insight into the mechanism behind the transcriptional induction of category 1 and 2 genes

Nuclear genes encoding photosynthesis-associated proteins are regulated through complex networks involving photoreceptors, circadian clock components and retrograde chloroplast signals of different origins [16], [73], [74], [75], [76]. These regulatory networks are only partly understood in green algae and higher plants [74], [75] and are almost completely unknown in diatoms. The differences in gene expression as a response to the different light quality conditions after D48 treatment are distinct at the 0.5 h time point for both the category 1 and 2 group of genes. With the experimental setup synchronizing the cell cycle, providing equal amount of PUR available for photosynthesis and thereby keeping the influence from plastidial signals similar for all light treatments, it seems likely that photoreceptor-mediated signals might be the cause of the observed initial differences when returned to light. With the exception of the category 2 gene *CPF2*, both confirmed and putative BL and RL photoreceptor genes are transcribed in D48 cells. The fast and light quality-dependent transcriptional response to exposure to light of different wavebands after D48 treatment indicates that not only are genes encoding photoreceptors transcribed in darkness, but photoreceptors are also present in darkness as functional molecules capable of altering the transcriptional activity of the cells. Unfortunately, the expression profiles of the photoreceptor genes do not contribute much to the elucidation of their possible roles as transcriptional regulators. In order to separate between gene induction/repression caused by photoreceptor signals and chloroplast signals resulting from photosynthetic activity, linear PET was blocked by the addition of DCMU prior to 0.5 h exposure to different qualities of light. The combined results from the gene expression analysis with and without the inhibitor of photosynthesis confirm the complexity of the regulation of PhANG expression, and enable speculations about gene induction and repressor mechanisms in diatoms.

Two different hypothetical mechanisms for induction and repression of category 1 genes can be deduced from the experimental results. One model that can explain the early and strongest induction of PhANGs defined as category 1 genes [45] in RL and GL is that these genes are initially activated by the onset of light through a mechanism involving a retrograde chloroplast signal resulting from photosynthetic activity. The lower initial expression of the category 1 genes in BL might indicate that these genes are subjected to transient repression when experiencing sudden exposure to BL. The significantly lower expression of category 1 genes in BL, GL and RL exposed cultures where linear PET has been blocked (Figure 4A) might indicate that the category 1 gene induction is highly dependent of a chloroplast signal of unknown origin. The *P. tricornutum* genome encodes two putative phytochromes (Dph1 and SKP3) with unknown function [6], but gene induction in RL seems to be solely dependent on a chloroplast signal for all but one of the examined category 1 genes (Figure 4A). GL, on the other hand, is able to cause a moderate rise in expression level for some of the examined category 1 genes (e.g. *CHLG*, *LHCR1*, *LHCR4* and *OEE3*), despite the absence of a putative chloroplast signal. Considering that no GL receptor has ever been identified in diatoms [6], [25], this finding is both surprising and greatly intriguing. This might indicate that the diatom genome contains a novel photoreceptor capable of GL absorption, or that some of the already known photoreceptors are capable of absorbing photons emitted by the light source that are in the blue-green range (Figure S3A). Regardless of the GL sensory mechanism, the results from the DCMU+GL treatment suggest the existence of an additional activator of the category 1 genes that is independent of photosynthetic events. The microarray results showed BL to be a weaker initial inducer of category 1 genes than GL and RL. The DCMU+BL treatment caused expression levels not only to remain at D48 levels but also to fall beneath this low level for a few of the examined category 1 genes (Figure 4A). These results suggest that a BL photoreceptor might function in repression of the category 1 genes. The repression could be direct or indirect by inducing genes that encode proteins involved in photoprotection, which might somehow be incompatible with a simultaneous induction of genes associated with light harvesting and photosynthesis. A second explanation for the observed lower expression of the category 1 genes in DCMU-treated samples might also exist. A side effect of DCMU+light treatment is an increase in singlet oxygen (^1^O_2_) production originating from triplet Chl formation in PSII [77]. Increased amounts of ^1^O_2_ might be perceived by the algae as being exposed to HL, thereby initiating a signaling cascade that represses the induction of the category 1 genes. If this scenario is true, the weaker induction or lack of induction of gene transcription observed in DCMU+light samples is caused not just by the removal of a positive signal, but also by the formation of a negative one.

Regardless of which light quality the cultures were exposed to, blocking of the linear photosynthetic electron flow by the addition of DCMU led to lower category 1 gene transcript levels than in the corresponding controls without DCMU. In contrast, the transcriptional responses of the category 2 genes to inhibitor treatment were light quality specific. Removing the ability to perform photosynthesis had practically no effect on the strong induction of the category 2 genes in BL (Figure 4B), it caused slightly increased expression of a subset of these genes in GL and reduced the amount of category 2 gene transcript to D48 levels in RL. Our results indicate that several signals affect the expression of the category 2 genes, but that these genes are induced mainly through a signal mediated by a BL-absorbing photoreceptor. Silencing of *AUREO1* has been connected to the induction of HL acclimation [62], and overexpression of *CPF1* leads to reduced expression of the *LHCX1-3* genes [63], making these photoreceptors unlikely candidates for the induction of the category 2 genes. In contrast, silencing of *CRYP* leads to a significant reduction of LHCX protein levels [64], suggesting that the presence of this blue light receptor is connected to the expression of photoprotective LHCs. Even though our results imply that photoreceptor signaling is crucial for the induction of the category 2 genes, transcript levels of *LHCX2* (and also *LHCX1*) increased as a result of artificially reducing the PQ pool during LL, thereby mimicking HL conditions [76]. In contrast, artificially oxidation of the PQ pool did not manage to prevent the expression of these genes in HL [76]. The explanation might be that the signal originating from the redox state of the PQ pool is overridden by photoreceptor signaling, and possibly also by another plastidial signal originating from elevated levels of ^1^O_2_ [76]. Increased levels of ^1^O_2_ have been found to induce stress responses in plants [78], [79]. GL caused a much lower induction of the category 2 genes than BL, but increased transcriptional levels were observed when DCMU was added prior to the GL treatment. This observation can possibly be explained by an increased ^1^O_2_ formation in the DCMU+light-treated samples. As suggested above, formation of ^1^O_2_ might be perceived by the diatom as a signal of HL, and therefore contribute to the induction of the category 2 genes. However, expression of the category 2 genes were not elevated by the addition of DCMU prior to BL and RL treatment, even though similar amounts of ^1^O_2_ theoretically should be produced in the chloroplast of these cells as well. One reason might be that the category 2 genes are already maximally expressed in BL, so that an increased formation of ^1^O_2_ is not able to further increase the expression of these genes. In contrast to the unaffected or increased gene expression caused by BL or GL exposure of DCMU-treated cultures, addition of DCMU prior to RL exposure caused the expression of the category 2 genes to remain at D48 levels. This observation indicates that the low induction of a few of the category 2 genes in RL-treated cultures is independent of both photoreceptor signaling and increased levels of ^1^O_2_, but is likely to depend on a chloroplast signal resulting from photosynthetic activity.

### Is there a form of chromatic acclimation in diatoms?

The total cell pigment concentration decreased 20–40% from the CWL to the D48-treatment (Figure 5). During this process, the Fuco fraction increased while the Diadino fraction decreased, indicating that maximum light-harvesting capabilities after re-exposure to light is of primary importance to the algae rather than light protection. During the gradual increase in total cellular pigment concentration from 0.5 to 24 h of light re-exposure, the Fuco:Diadino ratio changed again in favour of Diadino. Combined, the results suggest that maximum light-harvesting capabilities are prioritized when no light is available, while light protection potential is most important during light periods, independent of light quality.

In spite of the general, light-independent trend in cell pigment concentration, the scaled relative *in vivo* fluorescence excitation spectra produced from cultures grown under WL, BL, GL or RL displayed an interesting response to long-term exposure to light of fixed and limited wavebands. Spectra from cultures grown in 0.5 and 6 h of WL, BL, GL and RL displayed equal light ETE by the light-harvesting pigments to PSII reaction centre Chl *a*, within the entire PAR region (400–700 nm; Figure 6A and 6B). At first glance, this pointed to a light-harvesting apparatus equally efficient in transferring all the wavelengths of excitation light to Chl *a* independent of previous growth-light quality. However, after 24 h of light exposure a weak trend started to emerge, showing a positive correlation between growth light quality and ETE in the corresponding region of the PAR spectrum (Figure 6C). The trend became increasingly pronounced after 48 h (Figure 6D), which might be interpreted as chromatic acclimation. However, recent reports show that increased light intensity cause a shift from Fuco_red_ enrichment in LL to Fuco_blue_ in HL [14]. This was indeed confirmed by the ETE response to LL-HL shift (Figure 7), which was identical to the ETE pattern of the 24–48 h BL samples at the corresponding time-points (Figure 6C and 6D). The observed changes in ETE efficiency observed in RL, is therefore most likely a result of LL acclimation of the FCP trimer composition due to absence of BL. Regardless of the mechanism behind the adjustment of the Fuco_red_:Fuco_blue_ ratio, the functional effect is enhanced ETE in the green/red waveband of the PAR spectrum in GL and RL-grown cells.

### Indications of a light quality-dependent ability of photoprotection and repair of photodamaged PSII

The microarray analyses generate transcriptome snapshots of cells exposed to WL, BL, GL and RL during the first 24 h of light exposure after D48 treatment, and provide, in combination with the gene expression data from the inhibitor study, mechanistic insight and valuable clues to how the different light regimes affect the algae. However, the exact amount, stability and activity of the proteins encoded by the genes discussed in this study cannot be extrapolated directly from the transcriptional data. Indications regarding the effect of differential regulation of the category 2 genes at the physiological level are given by the measurements of Φ_PSII_max_. WL and GL treatments caused intermediate levels of Φ_PSII_max_, while BL and RL represented the extremes in the high end and low end respectively (Figure 8). Consequently, the Φ_PSII_max_ in the BL and RL-treated cells are discussed in relation to their expression of the category 2 genes. The low initial Φ_PSII_max_ measured in the BL cultures might be associated with initiation of photoprotective mechanisms. The high BL-induced expression of *LHCR6*, *LHCR8*, *LHCR10* and *LHCX2-3*, which previously have been reported to be associated with HL responses [45], [46], [49], [50], [80], in combination with the detection of small amounts of Diato essential for NPQ [81], support this hypothesis. The decrease in Φ_PSII_max_ levels measured in RL cultures during the length of the experiment might be explained by a possible absence of protection against sudden exposure to RL, as suggested by the lack of induction of category 2 genes, as well as the much lower expression of genes encoding PSII repair proteins. Whereas prolonged RL exposure causes impaired photosynthetic performance of the cells, Φ_PSII_max_ increased with BL exposure time (Figure 8). This trend is in accordance with the results reported by Schellenberger Costa et al. [42], where *P. tricornutum* was found to perform equally well (measured as oxygen-based P_max_) in BL and RL during LL conditions, but lacked the ability to acclimate to higher intensity RL, for which BL was necessary. However, in a subsequent study by the same group [62], it was shown that in transgenic cell lines with reduced levels of AUREO1a, HL acclimation (measured as a reduction of cellular Chl *a* content, increased photosynthetic rates and photoprotective potential) could also be induced in RL. In our experiment, BL causes lower levels of AUREO1a transcripts than RL, which might contribute to the HL acclimation features of the BL-exposed cells. In a recent study by Jungandreas et al. [43] it was confirmed that photosynthesis rates are very similar in cells acclimated to low BL or low RL using light/dark cycles, and also in cells shifted from low BL to low RL or *vice versa*. Light quality shifts do however cause a metabolic reorganization of the cells. Transferring the cells from RL to BL induce an increase in protein production, whereas a BL to RL shift causes accumulation of carbohydrates [43]. If light quality is directly responsible for regulation of activity of metabolic enzymes, and also for the regulation of *de novo* synthesis of these enzymes, similar differences in protein and carbohydrate accumulation might also be present between cells exposed to BL or RL after prolonged dark treatment.

Even though light of all intensities damage the PSII reaction centre protein D1 (encoded by *PsbA*), thereby causing inactivation of PSII complexes [82], the moderate light intensities used in this study was not expected to cause damage to the PSII complex that would exceed the capacity of repair. Cells exposed to prolonged white light exposure (48 h; 500 µmol m^−2^ s^−1^) of much higher intensities than used in this study (500 µmol m^−2^ s^−1^ WL correspond to ∼1150 µmol m^−2^ s^−1^ RL), showed only a moderate drop in Φ_PSII_max_ during the first 12 h of HL exposure, and recovered to the initial high levels within the following hours (12–48 h) [46]. However, the observed decline in Φ_PSII_max_ with longer RL exposure times (6–96 h), seen in context with the lack of initial induction of photoprotection by Diato and PSII repair genes, indicated that long-term RL-exposed cells had an increased susceptibility to photodamage and/or a low PSII repair capacity. This explanation is strongly supported by the results from the high intensity RL_max_ experiment.

The results from the transcriptional analyses of RNA isolated from *P. tricornutum* cultures exposed to RL_max_ intensities for 0.5 h confirmed that the diatom is unable to boost the transcription of the category 2 genes in RL, even when exposed to high intensities. The severe and persistent decline in Φ_PSII_max_ during the 0.5–96 h of RL_max_ exposure (Figure 10A) confirmed the negative effect RL has to the photosynthetic machinery of *P. tricornutum*, and strongly implies that the protein products of the category 2 genes (Figure 4B) are necessary for optimal function of the photoprotection and PSII repair mechanisms. The barely detectable (<0.1) Φ_PSII_max_ at the 96 h time-point indicated a complete photo-inactivation of the PSII reaction centres [83] and/or cell death [84]. *De novo* synthesis of Diadino + Diato, which takes place in the presence of a transthylakoid proton gradient (ΔpH) [85], was substantial after 0.5 h of RL_max_ exposure (Figure 9). This was also reflected by the unusual detection of Neo in these cells, which represents the biosynthesis precursor of both Diadino and Fuco [48]. The NPQ value of 0.6 at this early time-point was comparable with the value based on measurements from our previous study on LL-HL shift [46]. The observed NPQ levels during RL_max_ resulted in a corresponding decrease in Φ_PSII_max_, as is usually seen during HL [86]. High irradiances will additionally cause increased production of ROS, independent of spectral quality. ROS can cause inactivation of PSII complexes by damaging the D1 protein directly, but also by restraining protein synthesis in the chloroplast and consequently translation of the D1 protein [82]. The ROS-scavenging antioxidant PRXQ is found to attach to PSII and is believed to have a specific function in protection of photosynthesis in higher plants [58]. In our study, *PRXQ* was transcribed at extremely low levels (close to background signal levels) in both moderate and high RL intensities. In contrast, *PRXQ* transcription was strongly induced by moderate BL exposure (Figure 3), and it was also found to be among the strongest and most consistently up-regulated genes after a LL to HL shift [46]. These observations suggest that the induction of *PRXQ*, as the other category 2 genes, depends not only on the light intensity but also on the quality of light.

A lack of induction of genes encoding proteins important for photoprotection and PSII repair might explain the high, though non-lethal levels of photodamage during the length of the RL_max_ experiment (96 h). Subsequent transfer to RL_low_ led to a surprising increase in photosynthetic efficiency already after 3 h (Figure 10A), while NPQ did not decrease significantly until the 24 h time-point of RL_low_ (Figure 10D). These results point to a functional, but low capacity PSII repair mechanism in RL. The genes encoding proteins for PSII repair mechanisms are moderately expressed during darkness and RL, which probably explains why the moderate RL-incubated algae in the study by Schellenberger Costa et al. [42] performed better during a RL day and night regime, as opposed to our growth regime of continuous RL.

In spite of an approximately equal amount of PUR in BL, GL and RL, the spectral quality of light influenced *P. tricornutum* responses to ambient light conditions. Light quality-independent chloroplast signaling combined with photoreceptor signaling is necessary for regulation of the expression of nucleus-encoded genes in order to achieve optimal acclimation of the photosynthetic apparatus to a changing light environment. Whereas cellular pigment concentrations seem to depend on the light intensity, light quality might influence the protein composition of the FCP complexes, leading to differential absorption properties of Fuco. The combined results from this study clearly show that diatoms are not adapted to acclimate to higher intensities of RL. The lack of evolution of RL-controlled mechanisms to prevent and repair photodamage is logical when seen in context with the properties of the underwater light field. In the natural aquatic environment, large amounts of RL will not be encountered without the simultaneous presence of high intensity BL.

## Material and Methods

Axenic *P. tricornutum* Bohlin clone Pt1 8.6 (CCMP632) was obtained from the culture collection of the Provasoli-Guillard National Center for Culture of Marine Phytoplankton, Bigelow Laboratory for Ocean Sciences, USA.

### Growth and experimental light conditions

Continuous, axenic culturing of *P. tricornutum* was done as described in Nymark et al. [46]. The cultures were incubated at 15°C under cool white fluorescent light (Philips TLD 36W/96; spectral distribution presented in Figure S3B providing a scalar irradiance (E_PAR_) of 100 µmol m^−2^ s^−1^ under continuous white light (CWL) conditions. The cells divided ∼1.5 day^-1^ under these conditions. Dark treatment of the cultures cause cell division to cease during the first 6 h, since *P. tricornutum* cells are arrested in the G1 phase when deprived of light [45], [87], [88]. Upon the onset of the experiment the cultures were therefore synchronized by a 48 h dark-treatment (D48) [87], [88]. Thereafter the algae were exposed to blue light (BL), green light (GL) or red light (RL) provided by a waveband specific LED panel (SL3500, Photon Systems Instruments; Figure S3A). The algae were exposed to 0.5 h, 6 h or 24 h of: 1) 230 µmol m^−2^ s^−1^ of RL, 2) 100 µmol m^−2^ s^−1^ of GL, 3) 50 µmol m^−2^ s^−1^ of BL or 4) 100 µmol m^−2^ s^−1^ of WL respectively. This distribution of wavelength-specific irradiance ensured an equal rate of photons for absorption by the algae and thus available for photosynthetic energy transfer, i.e. defined as the Photosynthetically Usable Radiation (PUR) [8]. In order to keep the cell division rate in the cultures relatively constant during the length of the experiment (1–3 days), we chose the RL intensity that resulted in this same cell division rate as in the initial CWL culture, which was ∼1.5 day^−1^. The intensities of the GL and BL were calculated to correspond to the same amount of absorbed quanta in PSII as the RL-treated cells, resulting in a cell division rate at ∼1.5 day^−1^ also under these light conditions. By giving the algae equal amount of PUR accessible to PSII, we aimed to investigate differences caused by the light quality alone.

### Irradiance measurements and calculations

Growth light was measured inside culture flasks filled with sterile seawater using a scalar (4π) irradiance sensor (QSL-100; Biospherical Instruments, San Diego, CA, USA). The spectral distribution of the growth and incubation light was measured using a spectroradiometer (RAMSES-ACC-VIS, TriOS, Oldenburg, Germany) from 400 to 700 nm with 1 nm resolution. For each colour treatment, i.e. BL, GL and RL, PUR was calculated by correcting the spectral distribution of the incubation LED light for the *in vivo* algal absorption spectra normalized to Chl *a* (black line in Figure S3A). Accordingly, the intensity of each LED was tuned to ensure that the same rate of photons was available for algal absorption at each colour treatment. The *in vivo* algae absorption spectra and the Chl *a* concentration were measured according to Hancke et al. [44].

### RNA isolation and processing

Harvesting of diatom cultures exposed to BL, GL or RL for 0.5 h, 6 h, or 24 h after D48 treatment and subsequent RNA isolation, quantification and verification of RNA integrity were performed as described in Nymark et al. [46].

### DNA microarray experiments

One-colour microarray experiments were performed as described in Nymark et al. [45] using 83–200 ng total RNA isolated from 27 samples (three biological replicates for each treatment and time-point) as starting material for the reverse transcription reactions.

### Statistical analyses of microarray data

The statistical analyses of the microarray data were performed as described in Nymark et al. [45]. The raw data resulting from the one-colour microarray experiments of RNA isolated from CWL, D48 and WL (0.5 h, 6 h, and 24 h) cultures presented in Nymark et al. [45] were included in the statistical analyses and normalization procedures. A design matrix was created and contrasts between WL and BL, GL and RL for each of the re-exposure time points (0.5 h, 6 h, or 24 h) were computed. The Benjamini and Hochberg's method was used to estimate the false discovery rate [89]. Genes with an adjusted p-value below 0.05 were considered to be statistically significant differentially expressed. The genes discussed in the text are represented with 2–5 different probes on each microarray. Presented expression ratios are an average of values obtained from the two probes closest to the 3′ end representing the genes in question. Supplementary information on the *P. tricornutum* genes discussed in the text is provided in Table S4.

### Quantitative real-time PCR

A two-step quantitative real-time PCR (qRT-PCR) was carried out on total RNA from WL samples in addition to the BL, GL and RL samples at the three different time points, altogether 36 samples (three biological replicates for each treatment and time-point). The qRT-PCR reactions were performed as described in Nymark et al. [46]. Forward and reverse primers used are listed in Table S5. LinRegPCR software [90], [91] was used to calculate mean PCR efficiency per amplicon and cycle threshold (Ct) values per sample. These data were imported into the qBase^PLUS^ software (Biogazelle), which calculated relative expression ratios (given as Calibrated Normalized Relative Quantities (CNRQ)) and performed statistical analyses on the results. The unpaired t-test was used to evaluate the significance of the estimated relative expression ratios. Genes with p-values below 0.05 were considered to be statistically significant differentially expressed. The *PsaA* gene (c4570-2312) encoding Photosystem I P700 chlorophyll *a* apoprotein A1 was used as a reference gene to calculate relative expression ratios of chloroplast-encoded target genes. For calculations of relative expression ratios of genes localized to the nucleus, two nucleus-encoded reference genes were utilized, the *RPS5* gene encoding 30S ribosomal protein S5 (Phatr2_42848) and a gene encoding a glycerol kinase (GK; Phatr2_50182). Both genes are represented by five different probes on the microarray, and showed no response to the different light treatments at any time point.

### Inhibitor study

To discriminate between photoreceptor mediated signals and chloroplast signals resulting from photosynthetic activity, the effect of blocking of the photosynthetic electron transport on gene expression was studied by performing an additional experiment. Initial growth conditions and experimental set up was kept as described above. 3-(3,4-dichlorophenyl)-1,1-dimethylurea (DCMU) was dissolved in ethanol to a stock concentration of 50 mM and added to the cultures (in complete darkness) at a final concentration of 50 µM (final concentration of ethanol 1‰) before exposing the cells to 0.5 h of BL, GL or RL. Identical amounts of ethanol were added to the control samples. D48 samples with and without the addition of DCMU were also included in the experiment. The DCMU-treated D48 samples were harvested 0.5 h after adding of the inhibitor and used to verify that the DCMU treatment itself did not affect gene expression levels. Harvesting of cells, total RNA isolation and gene expression analyses by qRT-PCR were performed as described above, including three biological replicates for both treated samples and control samples. Finding suitable reference genes that were unaffected by prolonged darkness, BL, GL, RL and the DCMU treatment was challenging, since these treatments have a profound impact on the global gene regulation. Expression of the *GK* gene and a series of other candidate reference genes were found to be affected by the DCMU+light-treatment. Consequently, only the *RPS5* gene was used for normalization of relative expression ratios in the inhibitor experiment. Only genes displaying an expression ratio (log 2 transformed)> +/−1.5 were therefore considered to be significantly regulated.

### Pigment analysis

The HPLC pigment analysis was performed according to Rodriguez et al. [92] using a Hewlett-Packard HPLC 1100 Series system. Pigment values from the HPLC analysis were calculated as femtomol (fmol) pigment per cell. Sampling and storage of the algal cultures was done as described previously [45]. Three biological replicates were used for each time-point and treatment. The BL, GL, RL (including maximum RL treatment (RL_max_, see below)) and WL experiments were conducted individually, each setup including CWL and D48 treatment and sampling prior to each colour incubation. The Student's t-test was performed to establish whether the cellular pigment concentrations of the different treatments and incubation times were significantly different at any of the time-points. P-values <0.05 were considered significant.

### Variable *in vivo* Chl *a* fluorescence (Pulse Amplitude Modulated fluorometry)

Variable *in vivo* Chl *a* fluorescence was measured as described in Nymark et al. [46]. Three biological replicates of all the light-treatments were used. Non-photochemical quenching (NPQ) during maximum red light (see below) was calculated from measured fluorescence yield, according to the Stern-Volmer equation (F_m_-F'_m_)/F'_m_.

### *In vivo* fluorescence excitation

*In vivo* fluorescence excitation spectra were measured as described in Nymark et al. [45]. Spectra were recorded in three biological replicates of CWL cultures, D48 cultures, and from the same cultures subsequently re-exposed to 0.5, 6 and 24 h of BL, GL, RL or the initial WL. Spectra of the five treatments at each time point were scaled to the red emission maximum of Chl *a* of the D48 culture. This was done to study the development of the light energy transfer efficiency (ETE) by the main photosynthetic pigments Chl *a*, Chl *c* and Fuco in the 400–500 nm range of the PAR spectrum, where they exhibit their maximum absorption [93]. Additional samples were incubated in 35 and 500 µmol m^−2^ s^−1^ of WL, which were the same intensities used in a previous paper on molecular responses to LL-HL shift [46]. This was done in order to compare the ETE response to differences in light intensity versus spectral light of equal PUR.

### Maximum red light (RL_max_: 730 µmol m^−2^ s^−1^) study

Additional samples were incubated in 730 µmol m^−2^ s^−1^ of RL (corresponding to 320 µmol m^−2^ s^−1^ of WL), which was the maximum possible intensity of the red diode light. The maximum red light (RL_max_) samples were incubated for 0.5 h and subsequently harvested for pigment analysis, a microarray screen (one sample), qRT-PCR analyses, *in vivo* absorption- and fluorescence excitation spectra and PAM analyses. *In vivo* fluorescence excitation spectra and PAM analyses were conducted through 96 h, after which the cells were monitored through a 72 h period of low RL (RL_low_) to investigate possible recovery. The RL_low_ intensity was set to 80 µmol m^−2^ s^−1^, corresponding to 35 µmol m^−2^ s^−1^ of WL, which in a previous paper was defined as LL [46].

## Supporting Information

Figure S1 Expression ratios for genes in the hypothesized carotenoid biosynthetic pathway in *P. tricornutum.*
(PDF)

Figure S2 Fractions of Chl *a*, Chl *c*, Fuco, Diadino and Diato of total cellular pigment concentrations in the 0.5–24 h WL, BL, GL and RL treated cells.
(PDF)

Figure S3 Spectral composition of the blue, green and red diode light and the white, fluorescent light source.
(PDF)

Table S1 Effect of light quality on chloroplast gene expression in cultures treated with light of different quality.
(DOC)

Table S2 Effect of DCMU on gene expression in cultures treated with light of different quality.
(DOC)

Table S3 Category 2 gene expression in RL_max_ treated cells compared to RL treated cells.
(DOC)

Table S4 Information on *P. tricornutum* genes discussed in the text.
(DOC)

Table S5 Primers used for quantitative real-time PCR.
(DOC)
