# Supplementary material for: System Responses to Equal Doses of Photosynthetically Usable Radiation of Blue, Green, and Red Light in the Marine Diatom Phaeodactylum tricornutum
Source: PLoS One. 2014 Dec 3;9(12):e114211. doi: 10.1371/journal.pone.0114211 (PMC4254936; doi:10.1371/journal.pone.0114211)
Supplement: Figure S1 — Expression ratios for genes in the hypothesized carotenoid biosynthetic pathway in P. tricornutum. (PDF) [file pone.0114211.s001.pdf]

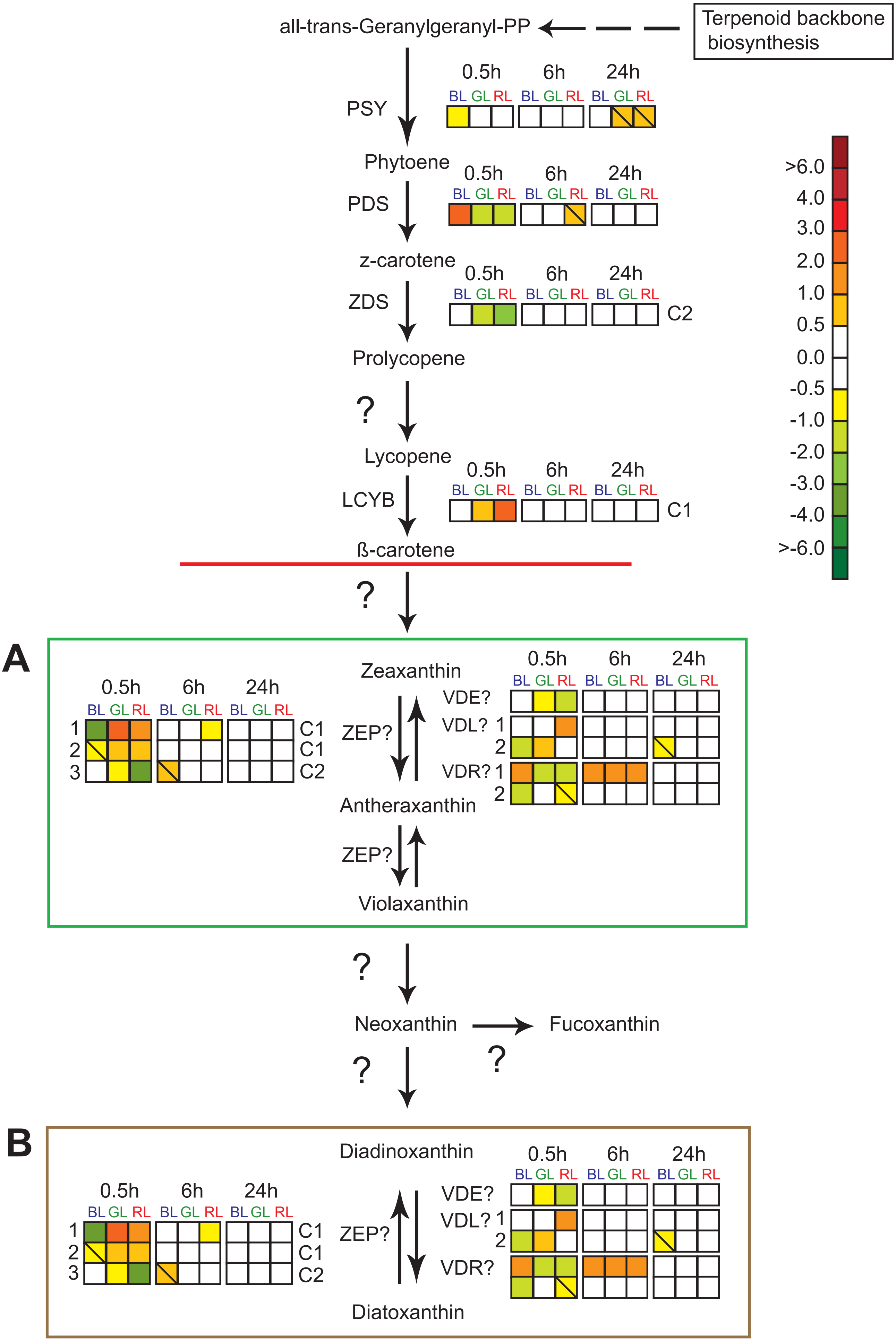

all-trans-Geranylgeranyl-PP
Terpenoid backbone biosynthesis
PSY
Phytoene
PDS
z-carotene
ZDS
Prolycopene
?
Lycopene
LCYB
ß-carotene
0.5h
6h
24h
BL GL RL
C1
C2
>6.0
4.0
3.0
2.0
1.0
0.5
0.0
-0.5
-1.0
-2.0
-3.0
-4.0
>-6.0
A
Zeaxanthin
ZEP?
VDE?
VDL?
VDR?
Antheraxanthin
Violaxanthin
Neoxanthin
Fucoxanthin
B
Diadinoxanthin
Diatoxanthin
