# Supplementary figures and images for: System Responses to Equal Doses of Photosynthetically Usable Radiation of Blue, Green, and Red Light in the Marine Diatom Phaeodactylum tricornutum
Source: PLoS One. 2014 Dec 3;9(12):e114211. doi: 10.1371/journal.pone.0114211 (PMC4254936; doi:10.1371/journal.pone.0114211)

**A**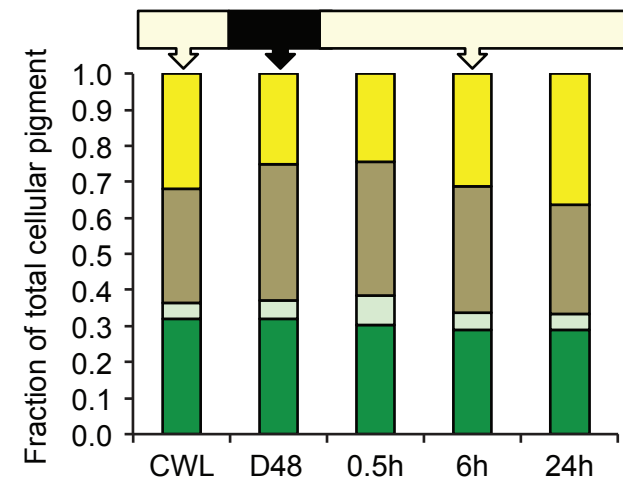**B**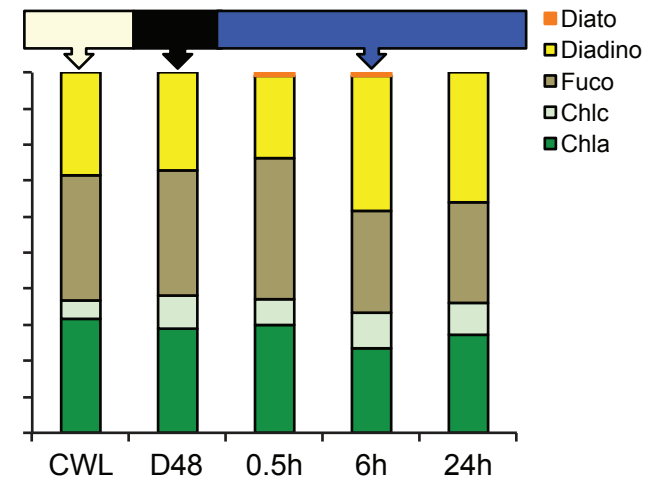**C**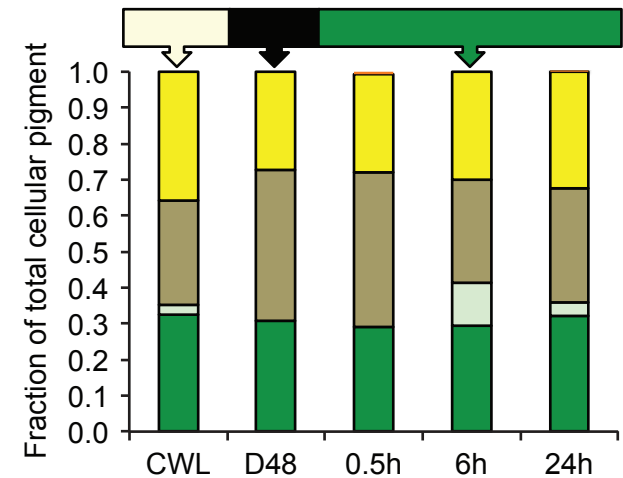**D**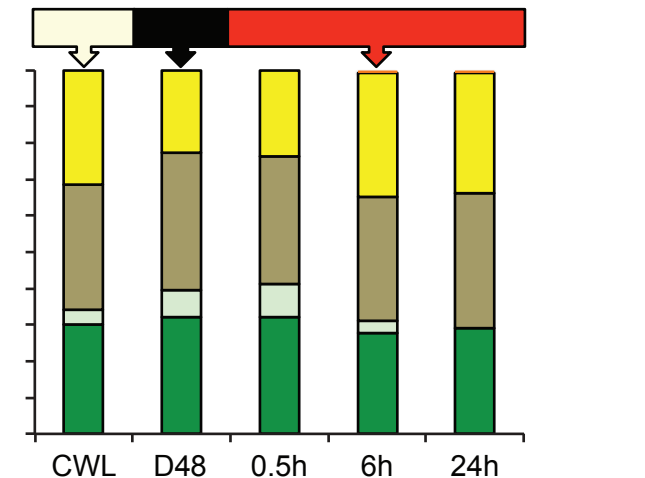

Supplement: Figure S2 — Fractions of Chl a, Chl c, Fuco, Diadino and Diato of total cellular pigment concentrations in the 0.5–24 h WL, BL, GL and RL treated cells. (PDF) [file pone.0114211.s002.pdf]

**A**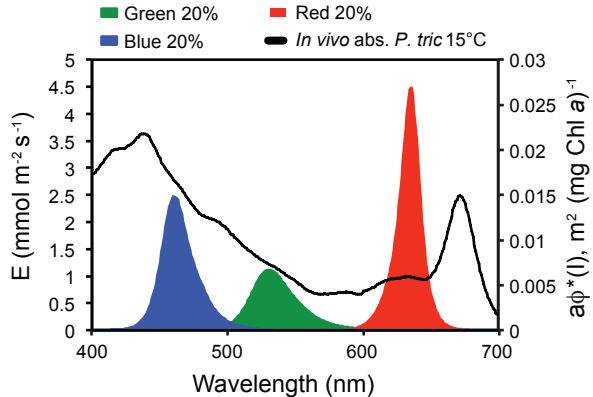**B**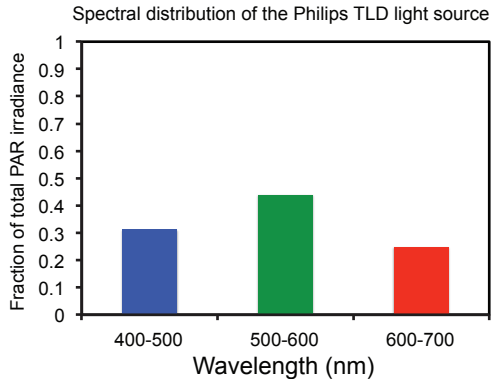

Supplement: Figure S3 — Spectral composition of the blue, green and red diode light and the white, fluorescent light source. (PDF) [file pone.0114211.s003.pdf]
