# Supplementary material for: System Responses to Equal Doses of Photosynthetically Usable Radiation of Blue, Green, and Red Light in the Marine Diatom Phaeodactylum tricornutum
Source: PLoS One. 2014 Dec 3;9(12):e114211. doi: 10.1371/journal.pone.0114211 (PMC4254936; doi:10.1371/journal.pone.0114211)

**Supplemental Table 1. Effect of light quality on chloroplast gene expression in cultures treated with light of different quality.**

Relative expression ratios (log_2_ transformed) for photosynthesis-related chloroplast genes were calculated by A) qRT-PCR analyses and B) microarray analyses by comparing the gene expression in cultures exposed to BL, GL or RL for 0.5 h, 6 h and 24 h versus the expression in WL-treated cultures at corresponding time points. Expression ratios calculated from the microarray analyses are an average of values obtained from the two probes closest to the 3’ end representing the genes in question. Significantly regulated genes are marked with V (qRT-PCR: p-value < 0.05 (student’s t-test); microarray: p-value < 0.01 (Limma package and R).


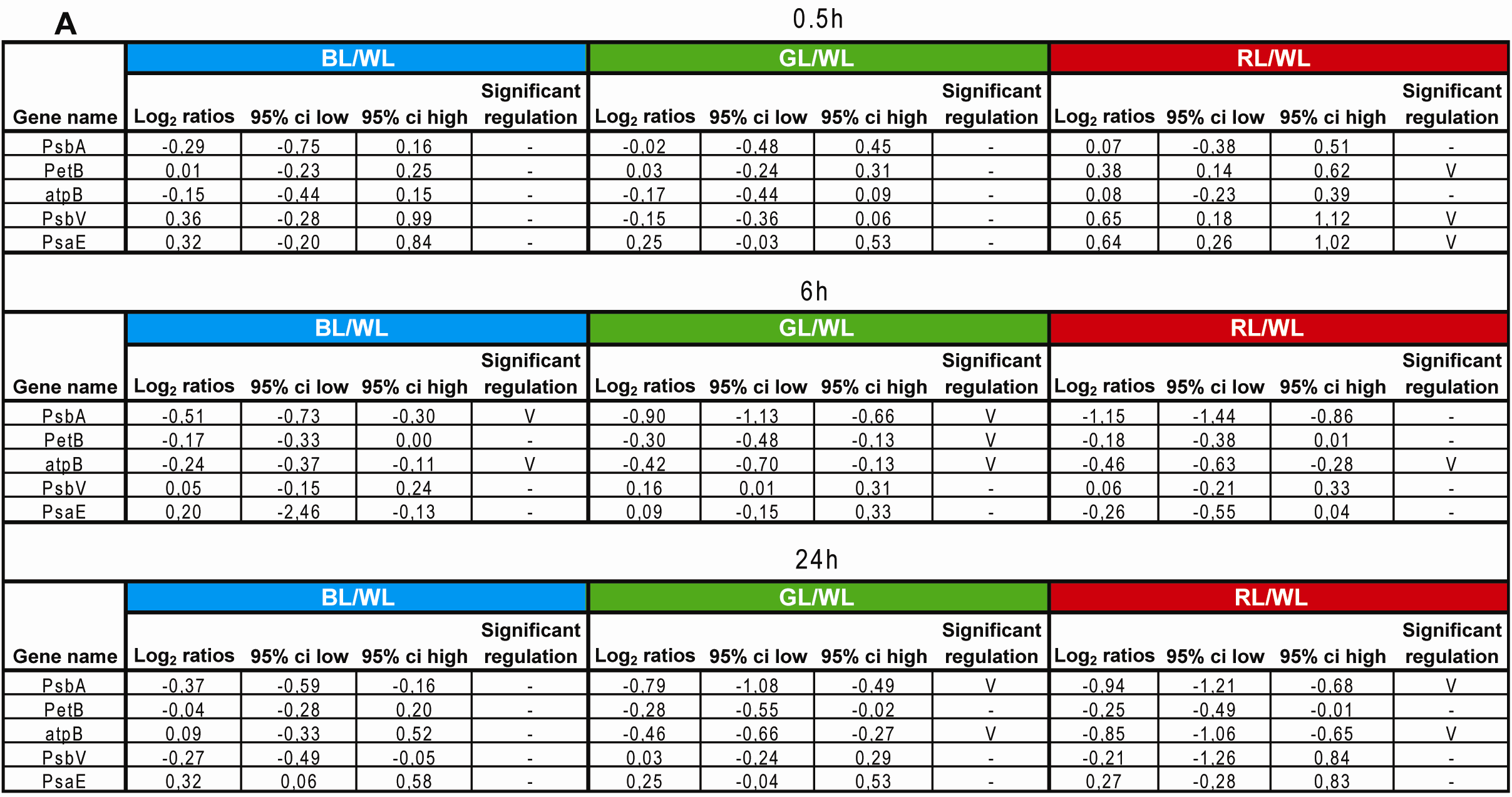


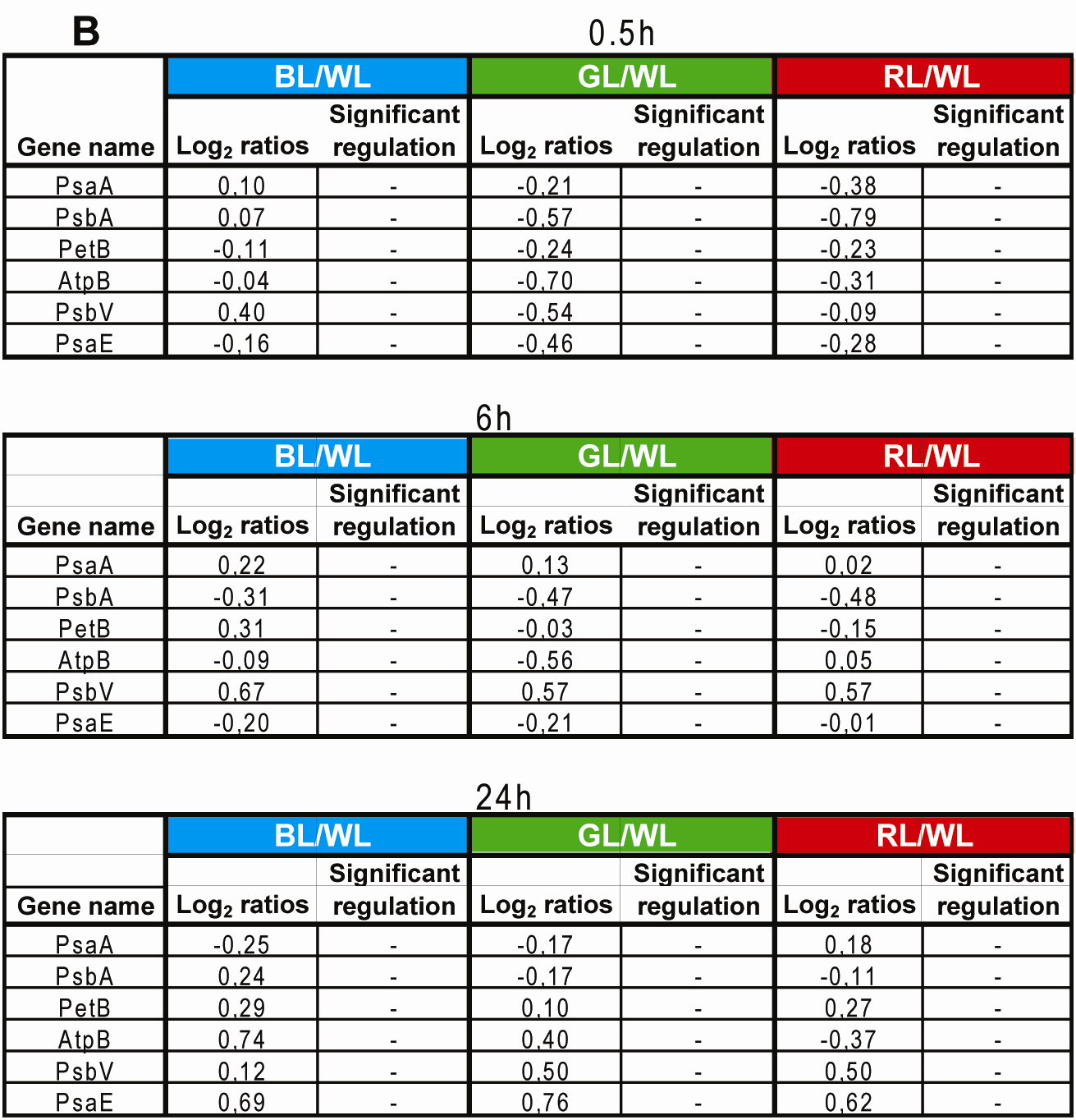

Supplement: Table S1 — Effect of light quality on chloroplast gene expression in cultures treated with light of different quality. (DOC) [file pone.0114211.s004.doc]
