# Supplementary material for: System Responses to Equal Doses of Photosynthetically Usable Radiation of Blue, Green, and Red Light in the Marine Diatom Phaeodactylum tricornutum
Source: PLoS One. 2014 Dec 3;9(12):e114211. doi: 10.1371/journal.pone.0114211 (PMC4254936; doi:10.1371/journal.pone.0114211)

**Supplemental Table 2. Effect of DCMU on gene expression in cultures treated with light of different quality.**

Relative gene expression ratios (log_2_ transformed) were calculated by qRT-PCR analyses of the expression of a subset of category 1 and 2 genes in DCMU+light treated (BL, GL or RL) cultures versus the expression in corresponding control cultures without the photosynthetic inhibitor. DCMU was added in complete darkness. The light exposure time was 0.5 h. Genes showing a relative expression ratio > +/- 1.5 and a p-value < 0.05 (student’s t-test) were considered to be significantly regulated (marked with V).


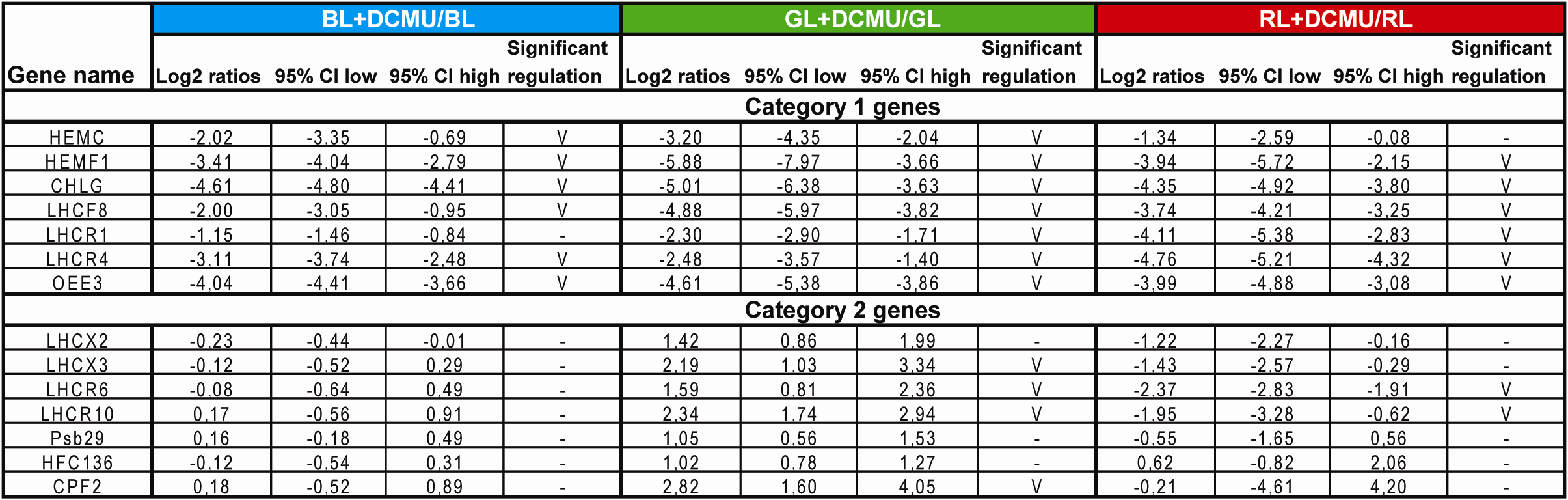

Supplement: Table S2 — Effect of DCMU on gene expression in cultures treated with light of different quality. (DOC) [file pone.0114211.s005.doc]
