# Supplementary material for: System Responses to Equal Doses of Photosynthetically Usable Radiation of Blue, Green, and Red Light in the Marine Diatom Phaeodactylum tricornutum
Source: PLoS One. 2014 Dec 3;9(12):e114211. doi: 10.1371/journal.pone.0114211 (PMC4254936; doi:10.1371/journal.pone.0114211)

**Supplemental Table 3. Category 2 gene expression in RL_max_ treated cells compared to RL treated cells.** Relative gene expression ratios (log_2_ transformed) were calculated by qRT-PCR analyses of the expression of a subset of category 2 genes in 0.5 h RL_max_ treated cells versus 0.5 h RL treated cells.


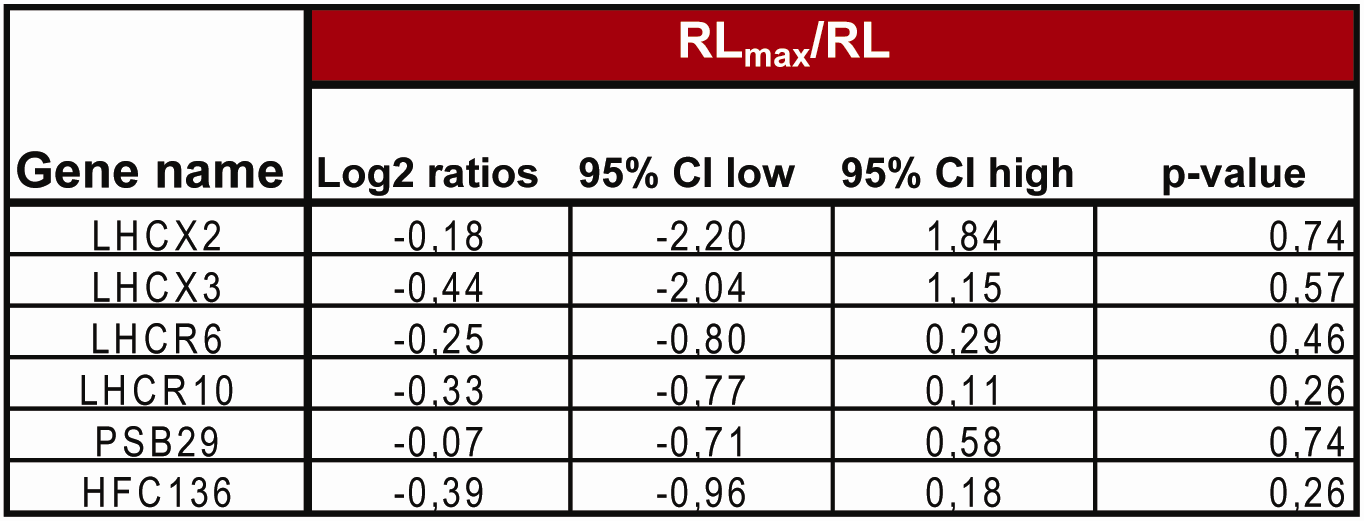

Supplement: Table S3 — Category 2 gene expression in RLmax treated cells compared to RL treated cells. (DOC) [file pone.0114211.s006.doc]
