# Supplementary material for: System Responses to Equal Doses of Photosynthetically Usable Radiation of Blue, Green, and Red Light in the Marine Diatom Phaeodactylum tricornutum
Source: PLoS One. 2014 Dec 3;9(12):e114211. doi: 10.1371/journal.pone.0114211 (PMC4254936; doi:10.1371/journal.pone.0114211)
Supplement: Table S4 — Information on P. tricornutum genes discussed in the text. (DOC) [file pone.0114211.s007.doc]

| **Supplemental Table 4. Information on *P. tricornutum* genes discussed in the text.** | | |  |  |  |
| --- | --- | --- | --- | --- | --- |
| Parameters given are the protein identification number (ID) and the NCBI accession numbers. Genes displaying the category 1 or 2 expression | | | | | |
| profile as a response to treatment with light of different quality (WL, BL, GL or RL) are indicated with 1 or 2, respectively. The division into | | | | | |
| category 1 (subcategories I-III), 2 and 3 from Nymark et al. (2013) are also indicated in the table. | | | |  |  |
|  |  |  |  |  | **Gene expression patterns** |
|  |  |  |  | **Expression profile** | **supporting the previous** |
|  |  |  | **Protein ID** | **category from** | **division into category** |
| **Protein** | **Abbreviation** | **Accession** | **Phatr2** | **Nymark et al. (2013)** | **1 or 2.** |
| glutamyl-tRNA synthetase | GLURS_1 | XP_002177350 | 51430 | 2 | - |
| glutamyl-tRNA synthetase | GLURS_2 | XP_002184854 | 52655 | - | - |
| glutamyl-tRNA reductase | HEMA | XP_002177785 | 54134 | - | - |
| glutamate-1-semialdehyde 2,1-aminomutase | HEML | XP_002180967 | 36347 | 1_I | 1 |
| porphobilinogen synthase | HEMB | XP_002177697 | 41746 | 1_II | 1 |
| hydroxymethylbilane synthase | HEMC | XP_002179459 | 51811 | 1_I | 1 |
| uroporphyrinogen-III synthase | HEMD | XP_002178352 | 44610 | - | - |
| uroporphyrinogen decarboxylase | HEME | XP_002184319 | 16140 | 1 | 1 |
| uroporphyrinogen decarboxylase | HEME_1 | XP_002180946 | 20757 | - | - |
| uroporphyrinogen decarboxylase | HEME_2 | XP_002178653 | 19188 | - | - |
| coproporphyrinogen III oxidase | HEMF_1 | XP_002182874 | 15068 | 1_I | 1 |
| coproporphyrinogen III oxidase | HEMF_2 | XP_002186510 | 10640 | 1_I | 1 |
| coproporphyrinogen III oxidase | HEMF_3 | XP_002179603 | 12186 | - | - |
| protoporphyrinogen oxidase | PPO | XP_002185274 | 31109 | 1_II | 1 |
| Protoporphyrin IX magnesium chelatase, subunit H | CHLH_1 | XP_002185833 | 13265 | - | - |
| Magnesium-chelatase, subunit H | CHLH_2 | XP_002177975 | 10100 | 3 |  |
| Protoporphyrin IX magnesium chelatase, subunit D | CHLD | XP_002178119 | 33017 | 1_II | 1 |
| magnesium-chelatase subunit I | ChlI | YP_874390 | - | 1_I | - |
| Magnesium-protoporphyrin IX methyltransferase | CHLM | XP_002178460 | 18872 | 1_I | 1 |
| NADPH:protochlorophyllide oxidoreductase A | POR1 | XP_002179689 | 12155 | - | - |
| NADPH:protochlorophyllide oxidoreductase A | POR2 | XP_002180992 | 13001 | - | - |
| NADPH-protochlorophyllide oxidoreductase | POR3 | XP_002177468 | 43164 | - | - |
| NADPH-protochlorophyllide oxidoreductase | POR4 | XP_002178962 | 34307 | 2 | - |
| 3,8-divinyl protochlorophyllide a 8-vinyl reductase | DVR | XP_002184654 | 30690 | 1_I | 1 |
| chlorophyll synthetase | CHLG | XP_002180392 | 12807 | 1_I | 1 |
|  |  |  |  |  | **Gene expression patterns** |
|  |  |  |  | **Expression profile** | **supporting the previous** |
|  |  |  | **Protein ID** | **category from** | **division into category** |
| **Protein** | **Abbreviation** | **Accession** | **Phatr2** | **Nymark et al. (2013)** | **1 or 2.** |
| 1-deoxy-d-xylulose-5-phosphate synthase | DXS | XP_002176386 | 46608 | - | - |
| 1-deoxy-D-xylulose-5-phosphate reductoisomerase | DXR | XP_002176954 | 9258 | - | - |
| 2-c-methyl-d-erythritol 4-phosphate cytidylyltransferase | ISPD_1 | XP_002182465 | 14857 | - | - |
| 2-c-methyl-d-erythritol 4-phosphate cytidylyltransferase | ISPD_2 | XP_002182074 | 21829 | - | - |
| 4-diphosphocytidyl-2c-methyl-d-erythritol kinase | CMK | XP_002178363 | 51700 | 1_II | 1 |
| 2C-methyl-D-erythritol 2,4-cyclodiphosphate synthase | ISPF | XP_002180037 | 12330 | - | - |
| 1-hydroxy-2-methyl-2-(E)-butenyl-4-diphosphate synthase | HDS | XP_002179061 | 44955 | - | - |
| hydroxymethylbutenyl diphosphate reductase | HDR | XP_002178617 | 41845 | - | - |
| isopentenyl-diphosphate delta-isomerase | IDI_1 | XP_002180259 | 12533 | 1_I | 1 |
| Isopentenyl-dephosphate delta-isomerase-like protein | IDI_2 | XP_002180941 | 12972 | - | - |
| farnesyl diphosphate synthase | FDPS | XP_002184024 | 49325 | - | - |
| geranylgeranyl pyrophosphate synthase | GGPS_1 | XP_002181666 | 47271 | - | - |
| geranylgeranyl pyrophosphate synthase | GGPS_2 | XP_002178555 | 19000 | 1_I | 1 |
| geranylgeranyl reductase | CHLP | XP_002176632 | 31683 | 1_I | 1 |
| fucoxanthin chlorophyll a/c protein | LHCF1 | XP_002177871 | 18049 | 1_I | 1 |
| fucoxanthin chlorophyll a/c protein | LHCF2 | XP_002177870 | 25172 | 1_III | 1 |
| fucoxanthin chlorophyll a/c protein | LHCF3 | XP_002177869 | 50705 | 1_I | 1 |
| fucoxanthin chlorophyll a/c protein | LHCF4 | XP_002177868 | 25168 | 1_I | 1 |
| fucoxanthin chlorophyll a/c protein | LHCF5 | XP_002184620 | 30648 | 1_III | 1 |
| fucoxanthin chlorophyll a/c protein | LHCF6 | XP_002182305 | 29266 | 1_II | 1 |
| fucoxanthin chlorophyll a/c protein | LHCF7 | XP_002184540 | 30643 | 1_III | 1 |
| fucoxanthin chlorophyll a/c protein | LHCF8 | XP_002182937 | 22395 | 1_I | 1 |
| fucoxanthin chlorophyll a/c protein | LHCF9 | XP_002183709 | 30031 | 1_I | 1 |
| fucoxanthin chlorophyll a/c protein | LHCF10 | XP_002182219 | 22006 | 1_III | 1 |
| fucoxanthin chlorophyll a/c protein | LHCF11 | XP_002184619 | 51230 | 1_III | 1 |
| fucoxanthin chlorophyll a/c protein | LHCF12 | XP_002184765 | 16302 | 1_I | 1 |
| fucoxanthin chlorophyll a/c protein | LHCF13 | XP_002183291 | 22680 | 1_III | 1 |
| fucoxanthin chlorophyll a/c protein | LHCF14 | XP_002186206 | 25893 | 1_II | 1 |
| fucoxanthin chlorophyll a/c protein | LHCF15 | XP_002183381 | 48882 | - | - |
|  |  |  |  |  | **Gene expression patterns** |
|  |  |  |  | **Expression profile** | **supporting the previous** |
|  |  |  | **Protein ID** | **category from** | **division into category** |
| **Protein** | **Abbreviation** | **Accession** | **Phatr2** | **Nymark et al. (2013)** | **1 or 2.** |
| fucoxanthin chlorophyll a/c protein | LHCF16 | XP_002178860 | 34536 | 1_I | 1 |
| fucoxanthin chlorophyll a/c protein | LHCF17 | XP_002184763 | 16322 | 1_II | 1 |
| fucoxanthin chlorophyll a/c protein | LHCR1 | XP_002178624 | 11006 | 1_II | 1 |
| fucoxanthin chlorophyll a/c protein | LHCR2 | XP_002183608 | 22956 | 1_II | 1 |
| fucoxanthin chlorophyll a/c protein | LHCR3 | XP_002178019 | 9799 | 1_I | 1 |
| fucoxanthin chlorophyll a/c protein | LHCR4 | XP_002177385 | 17766 | 1_I | 1 |
| fucoxanthin chlorophyll a/c protein | LHCR5 | XP_002182761 | 14986 | - | - |
| fucoxanthin chlorophyll a/c protein | LHCR6 | XP_002181976 | 14242 | 2 | 2 |
| fucoxanthin chlorophyll a/c protein | LHCR7 | XP_002177668 | 18180 | 2 | - |
| fucoxanthin chlorophyll a/c protein | LHCR8 | XP_002176917 | 32294 | 2 | 2 |
| fucoxanthin chlorophyll a/c protein | LHCR9 | XP_002186024 | 10243 | - | - |
| fucoxanthin chlorophyll a/c protein | LHCR10 | XP_002184869 | 16481 | 2 | 2 |
| fucoxanthin chlorophyll a/c protein | LHCR11 | XP_002184127 | 23257 | 1_II | 1 |
| fucoxanthin chlorophyll a/c protein | LHCR12 | XP_002176857 | 54027 | 1_I | 1 |
| fucoxanthin chlorophyll a/c protein | LHCR13 | XP_002182329 | 14442 | 1_I | 1 |
| fucoxanthin chlorophyll a/c protein | LHCR14 | XP_002182162 | 14386 | 1_I | 1 |
| fucoxanthin chlorophyll a/c protein | LHCX1 | XP_002179760 | 27278 | - | - |
| fucoxanthin chlorophyll a/c protein | LHCX2 | XP_002176987 | 54065 | 2 | 2 |
| fucoxanthin chlorophyll a/c protein | LHCX3 | XP_002178699 | 44733 | 2 | 2 |
| fucoxanthin chlorophyll a/c protein | LHCX4 | XP_002182760 | 38720 | - | - |
| fucoxanthin chlorophyll a/c protein | LHC6062 | XP_002182909 | 6062 | 1_I | 1 |
| fucoxanthin chlorophyll a/c protein | LHC13877 | XP_002181795 | 13877 | 1_I | 1 |
| fucoxanthin chlorophyll a/c protein | LHCZ1 | XP_002183911 | 15820 | - | - |
| fucoxanthin chlorophyll a/c protein | LHC17531 | XP_002176735 | 17531 | 1_II | 1 |
| fucoxanthin chlorophyll a/c protein | LHC24119 | XP_002185437 | 24119 | 1_II | 1 |
| fucoxanthin chlorophyll a/c protein | LHC48798 | XP_002183454 | 48798 | 1_II | 1 |
| red lineage chlorophyll a/b-binding-like protein | LHL1 (RedCAP) | XP_002177121 | 17326 | 1_III | 1 |
| photosystem I P700 apoprotein A1 | PsaA | YP_874359 | - | - | - |
| photosystem I reaction center subunit IV | PsaE | YP_874428 | - | - | - |
|  |  |  |  |  | **Gene expression patterns** |
|  |  |  |  | **Expression profile** | **supporting the previous** |
|  |  |  | **Protein ID** | **category from** | **division into category** |
| **Protein** | **Abbreviation** | **Accession** | **Phatr2** | **Nymark et al. (2013)** | **1 or 2.** |
| photosystem II reaction center protein D1 | PsbA | YP_874444 | - | - | - |
| Photosystem II reaction center M protein precursor | PSBM | XP_002184128 | 55057 | 1_II | 1 |
| oxygen-evolving enhancer protein 1 | PSBO | XP_002180309 | 20331 | 1_III | 1 |
| Photosystem II oxygen evolution complex protein PsbP | PSBP | XP_002182797 | 48359 | 2 | - |
| oxygen-evolving enhancer protein 3 | OEE3/PSBQ' | XP_002180307 | 54499 | 1_II | 1 |
| Photosystem II 12 kDa extrinsic protein (PsbU) | PSBU | XP_002178366 | 26293 | 1_II | 1 |
| photosystem ii cytochrome c550 | PsbV | YP_874401 | - | - | - |
| extrinsic protein in photosystem II | PSB31 | XP_002180868 | 46529 | 1_II | 1 |
| cytochrome b6 | PetB | YP_874393 | - | - | - |
| cytochrome b6-f complex iron-sulfur subunit | PETC | XP_002185823 | 46657 | 3 |  |
| cytochrome b6-f complex iron-sulfur subunit | PETC2 | XP_002185824 | 13358 | 1_I | - |
| ferredoxin-NADP reductase | PETH | XP_002184856 | 23717 | - | - |
| cytochrome c6 | PETJ | XP_002186138 | 44056 | 1_II | - |
| atp synthase cf1 alpha chain | AtpA | YP_874426 | - | - | - |
| ATPase gamma subunit | ATPC | XP_002180505 | 20657 | - | - |
| blue light receptor | AUREO1a | XP_002183783 | 8113 | - | - |
| blue light receptor | AUREO2 | XP_002183315 | 15468 | - | - |
| blue light receptor | AUREO1b | XP_002184107 | 15977 | - | - |
| blue light receptor | AUREO1c | XP_002180003 | 51933 | 2 | - |
| cryptochrome-like protein 2 | CRYL2 | XP_002179379 | 54342 | - | - |
| blue light photoreceptor | CPF1 | XP_002180095 | 27429 | - | - |
| CRY DASH-like protein | CPF2 | XP_002178889 | 34592 | 2 | 2 |
| similar to Cryptochrome DASH | CPF4 | XP_002184557 | 55091 | - | - |
| sensor kinase protein 3 | SKP3 | XP_002184053 | 55037 | 3 |  |
| phytoene synthase | PSY | XP_002178776 | 41878 | - | - |
| phytoene dehydrogenase | PDS1 | XP_002180171 | 35509 | - | - |
| zeta-carotene desaturase | ZDS | XP_002176685 | 9040 | 2 | 2 |
| chloroplast lycopene beta cyclase precursor | LCYB | XP_002176612 | 8835 | 1_I | 1 |
| zeaxanthin epoxidase | ZEP1 | XP_002180238 | 45845 | 1_I | 1 |
|  |  |  |  |  | **Gene expression patterns** |
|  |  |  |  | **Expression profile** | **supporting the previous** |
|  |  |  | **Protein ID** | **category from** | **division into category** |
| **Protein** | **Abbreviation** | **Accession** | **Phatr2** | **Nymark et al. (2013)** | **1 or 2.** |
| zeaxanthin epoxidase | ZEP2 | XP_002176935 | 5928 | 1_II | 1 |
| zeaxanthin epoxidase | ZEP3 | XP_002178367 | 10970 | 2 | 2 |
| violaxanthin deepoxidase | VDE | XP_002178643 | 51703 | - | - |
| violaxanthin deepoxidase-like protein | VDL1 | XP_002180635 | 36048 | - | - |
| violaxanthin deepoxidase-like protein | VDL2 | XP_002180051 | 45846 | - | - |
| violaxanthin deepoxidase-related | VDR1 | XP_002177513 | 43240 | - | - |
| violaxanthin deepoxidase-related | VDR2 | XP_002176267 | 42262 | - | - |
| photosystem II 11 kD protein | PSB27 | XP_002177207 | 9078 |  | 2 |
| PBS29/Thf1-like protein | PSB29 | XP_002182081.1 | 37959 |  | 2 |
| photosystem II stability/assembly factor HCF136 | HCF136 | XP_002181754 | 13895 |  | 2 |
| cell division protein FtsH | FTSH1 | XP_002177215 | 17504 |  | - |
| cell division protein FtsH | FtsH2 | YP_874427 | - |  | - |
| glutaredoxin | GLRXC2 | XP_002183091 | 39133 |  | 2 |
| peroxiredoxin Q | PRX Q | XP_002181851 | 21736 |  | 2 |
| GTP-binding protein TypA | TYPA1 | XP_002184547 | 40621 |  | - |
| GTP-binding protein TypA | TYPA2 | XP_002184487 | 23444 |  | - |
|  | | |  |  |  |
|  | | | | | |
|  | | | | | |
|  | | | |  |  |
|  |  |  |  |  |  |
|  |  |  |  |  |  |
|  |  |  |  |  |  |
