# Supplementary material for: System Responses to Equal Doses of Photosynthetically Usable Radiation of Blue, Green, and Red Light in the Marine Diatom Phaeodactylum tricornutum
Source: PLoS One. 2014 Dec 3;9(12):e114211. doi: 10.1371/journal.pone.0114211 (PMC4254936; doi:10.1371/journal.pone.0114211)
Supplement: Table S5 — Primers used for quantitative real-time PCR. (DOC) [file pone.0114211.s008.doc]

|  | | | |  |  | |  | |  |  |
| --- | --- | --- | --- | --- | --- | --- | --- | --- | --- | --- |
|  | | | |  |  | |  | |  |  |
| **Supplemental Table 5. Primers used for quantitative real-time PCR.** | | | | | |  | |  | | |
| **Gene** | **Accession** | **Orientation** | **Sequence** | | | **Amplicon size** | |  | | |
| PsaA | YP_874359 | forward | ATGCAGATGCTCATGACTTTGA | | | 91 | |  | | |
|  |  | reverse | TACTGATAACTGTCCAAAGTGC | | |  | |  | | |
| PsaE | YP_874428 | forward | ATTGGTTTAACGAAGTCGGAAC | | | 145 | |  | | |
|  |  | reverse | TGGACTGCTAACTTCAACTAAC | | |  | |  | | |
| PsbA | YP_874444 | forward | TGATATCGATGGTATTCGTGAG | | | 101 | |  | | |
|  |  | reverse | TGGATACCGATAGCATTAGAAC | | |  | |  | | |
| PsbV | YP_874401 | forward | TTAATGCAACTTGTGGTGCTTG | | | 121 | |  | | |
|  |  | reverse | AACCAAACCTGCTATGTTATCG | | |  | |  | | |
| PetB | YP_874393 | forward | GTTGTTGACGCATTTGCATCAG | | | 98 | |  | | |
|  |  | reverse | CACCATCATACTTGCTGACCAA | | |  | |  | | |
| AtpB | YP_874407 | forward | ATTCAAGCTGTATACGTACCAG | | | 70 | |  | | |
|  |  | reverse | TCTAAATGTGCGAATGTTGTCG | | |  | |  | | |
| chlI | YP_874390 | forward | GCAAATAGAGGACTACTTTACG | | | 98 | |  | | |
|  |  | reverse | AACTGTGTTCCAACCAGATGCT | | |  | |  | | |
| FtsH2 | YP_874427 | forward | GAAGTTGGTCATGCAATTGTCG | | | 109 | |  | | |
|  |  | reverse | TCTGGTGCAAACCATGTAAGAC | | |  | |  | | |
| OEE3 | XP_002180307 | forward | TGCGTTCTTCAAGGCTATCGAG | | | 142 | |  | | |
|  |  | reverse | GTCCAGGAGTCAGATGCAAATC | | |  | |  | | |
| PSB29 | XP_002182081 | forward | GGATGAAGTCTACCCCGTTATG | | | 126 | |  | | |
|  |  | reverse | CATGGTCTCCATCATGTCAAGC | | |  | |  | | |
| HFC136 | XP_002181754 | forward | ACTGGTGGATACGGCATTACTG | | | 93 | |  | | |
|  |  | reverse | CGTCAAAGCTCACGTACATCGT | | |  | |  | | |
| HEMC | XP_002179459 | forward | AGACTTCTGGACAGCAGGTTCT | | | 134 | |  | | |
|  |  | reverse | CCACGCGATGAATGTGATACCA | | |  | |  | | |
| HEMF1 | XP_002182874 | forward | ACCTCTAGTTACAGTATGACAG | | | 112 | |  | | |
|  |  | reverse | TGCCAACGAATGCTATTGTGCT | | |  | |  | | |
| CHLG | XP_002180392 | forward | AAGCCGTCTTTGGTACACTGGA | | | 75 | |  | | |
|  |  | reverse | ATTCCGAGTCCTGCAATTGAGT | | |  | |  | | |
| LHCX2 | XP_002176987 | forward | GCGCTCAAATTGGTTGGGTTGA | | | 98 | |  | | |
|  |  | reverse | GAGGATCAAAGCCAATGTCACC | | |  | |  | | |
| LHCX3 | XP_002178699 | forward | TTCTACAGACCAAGGAACTCCA | | | 130 | |  | | |
|  |  | reverse | TGTGCAAACGTTACGGGAAACT | | |  | |  | | |
| LHCF8 | XP_002182937 | forward | CTACCGGGAAACATTGACTACT | | | 75 | |  | | |
|  |  | reverse | TAATGGTATCCAGAGCCTTGAA | | |  | |  | | |
| LHC48798 | XP_002183454 | forward | CGGCTGGATTCTAAGTGACCTT | | | 139 | |  | | |
|  |  | reverse | TGTCTGTAGTCGCAGGCTATGT | | |  | |  | | |
| LHCR1 | XP_002178624 | forward | ACCTGGTAATCTTGGATTCGAC | | | 84 | |  | | |
|  |  | reverse | GATTTCCTTGAGTTGCATGGCT | | |  | |  | | |
| LHCR4 | XP_002177385 | forward | AAGCCAAGTATGTCGCCAGTGA | | | 70 | |  | | |
|  |  | reverse | ACCGACGTATCCTTTGAGGTTG | | |  | |  | | |
| LHCR6 | XP_002181976 | forward | CTTTCAGGAGTTCGTTTCCAAG | | | 77 | |  | | |
|  |  | reverse | TTAATGACGGTACCAATGGGCT | | |  | |  | | |
| LHCR10 | XP_002184869 | forward | CGGGAAATCTGAACTTTGATCC | | | 136 | |  | | |
|  |  | reverse | GATGGCTTCTTGAATGGCAAAC | | |  | |  | | |
| CPF2 | XP_002178889 | forward | GCTTTCCGCAAGCCAAAGTTCA | | | 103 | |  | | |
|  |  | reverse | CGTAAAGGTATCGGGCATGTTG | | |  | |  | | |
| RPS5 | XP_002176761 | forward | CGTTTCAAGATGGAAGCACTCA | | | 119 | |  | | |
|  |  | reverse | CGGAATGTAGTCAGTATCGTCA | | |  | |  | | |
| GK | XP_002185006 | forward | AGACGCCAGATACATCCAATCT | | | 73 | |  | | |
|  |  | reverse | TCAAGAAAGGTACCACCGTCAA | | |  | |  | | |
